# A course-undergraduate research experience (CURE) to explore the effect of structural variants on gene expression in *C. elegans* balancers

**DOI:** 10.1371/journal.pone.0342806

**Published:** 2026-08-11

**Authors:** Tatiana Maroilley, Victoria Rodrigues Alves Barbosa, Rumika Mascarenhas, Suzanne Ferris, Catherine Diao, Fatemah AlAwadhi, Saood Aldakheel, Ahmad Ali, Denah Alkanderi, Meshary Alshatti, Samyah Alsuwaileh, Ryan Bui, Bentley Chai, Lara Dsouza, Parisa Etemadi Nezhad, Emily Garcia-Volk, Zainub Haq, Sadeeb Hossain, Grace Johnson, Nidhi Kotikalapudi, Inara Lalani, Courtney Lenz, Taye Louie, Stephen Moore, Shubh Patel, Shuvam Prasai, Ronaar Qureshi, Farah Rahmani, Bilal Shakir, Sadhiya Siraj Ahamed, Hong Anh Tran, Radia Waziha, Cassandra M. Wood, Sara Zbinden, David Anderson, Maja Tarailo-Graovac

**Affiliations:** 1 Departments of Biochemistry, Molecular Biology and Medical Genetics, Cumming School of Medicine, University of Calgary, Calgary, Canada; 2 Alberta Children’s Hospital Research Institute, University of Calgary, Calgary, Canada; 3 Bachelor of Health Sciences, Cumming School of Medicine, University of Calgary, Calgary, Canada; Davidson College, UNITED STATES OF AMERICA

## Abstract

Bioinformatics, a discipline at the crossroads of Biology and Computational Sciences, also referred to as Computational Biology, is widely spread in research programs. However, implementing any Bioinformatics projects requires the ability to comprehend biological concepts and apply computational approaches. Yet, the undergraduate programs offering such multidisciplinary training are rare. In addition, understanding the interplay between Biology research projects and Bioinformatics analyses is challenging without hands-on experience. Course-based undergraduate research experience (CURE) courses are innovative programs that allow more students to acquire research experience and provide the perfect setting to introduce students to applied bioinformatics. As a part of the Bachelor of Health Sciences at the Cumming School of Medicine, University of Calgary (Canada), a CURE applied bioinformatics was implemented in the Winter semesters of 2023–2025. Students investigated the effect of structural variants (SVs, genetic variants larger than 50 bp) on gene expression in the model organism *Caenorhabditis elegans* (a hermaphrodite 1-mm long roundworm). The students detected and characterized SVs by analyzing genome and transcriptome sequencing data of *C. elegans* strains called balancers, as they are known to carry large genomic variations balancing regions of the genome by limiting recombination and allowing maintenance of lethal mutations. The students used Galaxy, a public web-based super-computing resource, but also a local High-Performance computing system, and R, to report different effects of SVs on gene expression and splicing. Beyond acquiring new skills, students uncovered new findings, such as effects of a reciprocal translocation *eT1(III;V)* on gene expression of *H14N18.2*. We obtained data on the course's impact on student learning journeys by administering a voluntary student-based survey with 14% response rate (standard response rate at the University of Calgary). Overall, the available data survey showed that CURE facilitated students understanding of the Bioinformatics field and fostered their research interest. Beyond these results, we provide here guidelines on facilitating similar CURE implementations to improve access to bioinformatics research experiences for undergraduate students.

## Introduction

For over a decade, innovative teaching methods have been implemented to increase interest for research, improve students’ learning experience, and allow the acquisition of a trans-disciplinary skillset [1–7]. In higher education, research-based teaching programs such as Course-based Undergraduate Research Experience (CURE) fostering active student-centered teaching methods, are flourishing in different fields [8–21], including Bioinformatics [22–28]. CUREs were originally inspired by apprenticeship, adapted to a classroom setting with a large audience [29]. The hallmarks of a CURE are: i) scientific process, ii) discovery, iii) relevance, iv) collaboration, and v) iteration. In CUREs, students apply scientific methods to authentic and novel research projects of interest for the scientific community [23]. CUREs are inquiry-based, which has been shown to improve student performance, their problem-solving and analytical skills, confidence, and increase their interest toward research [12,23,30–33]. For Instructors, CUREs are an opportunity to unite teaching and research activities. CUREs also provide an opportunity to develop and implement innovative teaching methods by reducing the amount of formal lectures and fostering mentorship activities [34,35].

Bioinformatics emerged originally as an interdisciplinary research field from the increasing need for Computer Sciences approaches in Life Sciences research, concomitant with the advent of high-throughput technologies and the development of high-performance computing systems. Consequently, Bioinformatics, also referred to as Computational Biology or Data Sciences, has been exponentially expanding requiring more and more high-qualified personals. However, with the field rapidly evolving, it has been a challenge to define it clearly, which may have led to limiting its attractiveness for undergraduate students. Furthermore, in Bioinformatics, Computational Biology, or Data Science, one obstacle to the implementation of CUREs has been the challenge for students to have prior pertinent knowledge in both Biology and Computational Sciences. This resulted in a perception of reduced accessibility for students and may cause anxiety for students who enrolled in a Bioinformatics class.

In addition, it required multi-disciplinary training with hands-on experience and knowledgeable instructors, access to which may not always be available to students. Indeed, as we entered the era of omics data, computational biologists had to adapt to different datasets (genomics, transcriptomics, metabolomics, proteomics…). But each type of data needed an understanding of the technologies used to acquire the data, and of the relevant molecular mechanisms. And, depending on the size of the dataset, mathematical modelling and statistical approaches might be necessary. Now, with the advent of artificial intelligence, even more advanced computational techniques will become routine in Bioinformatics in the years to come.

In the field of genomics, the advent of sequencing technologies has greatly improved our ability to explore genome variability. Structural variants (SVs) are defined as variations in the genome larger than 50 bp [36]. They can be balanced (translocations or inversions) or unbalanced (copy number variants such as deletions or duplications) [37]. In addition, complex genomic rearrangements (CGRs) are defined as an overlapping set of structural variants, impacting one or a few genomic loci. The most complex ones have been defined as chromoanagenesis (including chromothripsis, chromoanasynthesis, and chromoplexy), resulting from a catastrophic series of variations and DNA breakages re-shuffling entirely one or a few genomic regions [38]. Despite SVs and CGRs had been known to affect phenotypes, diseases, adaptation, and evolution [39], they remained understudied due to the challenges inherent to their detection. However, recent advances in high-throughput whole genome sequencing [39–45] or optical mapping technologies [46] have allowed the acquisition of datasets which when analyzed via tailored bioinformatics methods, enable accurate and efficient detection of SVs and CGRs. In fact, our team has previously reported over one hundred SVs and CGRs in balancer strains of the model organism *C. elegans* [47,48].

In clinical and biomedical studies, however, SVs and CGRs are still mostly underreported as the interpretation of their involvement in the pathogenic mechanisms is challenging [39]. For single-nucleotide variations and indels (small insertions or deletions < 50 bp), we mostly rely on *in silico* tools predicting the effect on genes or scoring their pathogenicity. Yes, when possible, the variant effects are tested by functional analyses via experimental approaches (model organism or measurement of intermediate phenotype such as RNA or protein levels [49,50]). However, *in silico* and experimental tools to predict and test the effect of SVs and CGRs are very limited.

For decades, like most Canadian post-secondary institutions, the University of Calgary (UCalgary) has innovated and improved education methods. In 2020, the College of Discovery, Creativity, and Innovation (CDCI) of the Taylor Institute for Teaching and Learning (TI) at UCalgary launched the Undergraduate Research Initiative (URI) to facilitate access to research experiences for undergraduate students and provide support to Instructors to develop and implement innovative teaching methods such as CURE. The Bachelor of Health Sciences (BHSc) program at the Cumming School of Medicine (UCalgary) is an inquiry-based research-intensive undergraduate degree program. Here, we present a CURE developed for the BHSc as an introduction to Bioinformatics, with the support of the TI, and implemented four times since Winter 2023, for over a hundred fifty students. We describe here the teaching methods and strategies we implemented with the hope that these could help guide others to develop similar programs. We also report on the results of a student survey that aimed to evaluate the experience students had with CURE and impacts on their learning journey. Furthermore, a research project led by the students assessed the original and authentic transcriptomics data aimed at exploring the functional impact of known SVs and CGRs found in the genomes of *C. elegans* balancer strains. We exemplify here the novel findings uncovered by our students and report on transcriptome results when the translocation *eT1(III;V)* was analyzed.

## Materials and Methods

### Overview

As part of the Bachelor of Health Sciences at the Cumming School of Medicine at the UCalgary, MDSC 301 is the introductory course to Bioinformatics that primarily serves second-year undergraduate students of the Bioinformatics program, but it is open to other programs. Original prerequisites for MDSC 301 were 6 units in Computer Science at the 300 level or Medical Science 341, or 6 units in Biological Sciences at the 300 level. The course had a 45-student capacity. In 2023, the course was redesigned as an applied bioinformatics CURE and then implemented four times. The MDSC 301 CURE has accommodated so far 153 students (Winter 2023 with 30 students, Winter 2024 with 35 students, Winter 2025 with 44 students, and Winter 2026 with 44 students). The CURE is implemented by a single instructor and one to three graduate assistants (a half Teaching Assistant, a Research Coach, and a Graduate Research Assistant) later referred to as TAs. The CURE unfolds over 12 weeks (January-April) and the class meets twice a week for 75 minutes. Classes alternate between short lectures and working sessions during which students work in groups and benefit from TAs and the instructor’s mentorship. The course is equipped with a computer science lab, portable wet-bench experimental material, web-based resources including publicly accessible servers (Galaxy), and local UCalgary high-performance computing systems dedicated to students. It is also supported by a molecular genetics research group (Dr. Tarailo-Graovac) and the TI. The main teaching material is in the form of a Lab Book, inspired by previously published CUREs [51]. This document is made available to the students on the first day of the semester and contains all the necessary information related to the CURE: templates, background information, CURE rules, guidelines, protocols, schedule, and scripts. As an example, the Lab Book 2024 can be found at https://github.com/MTG-Lab/MDSC301.

### Survey and Ethics statement

Students enrolled in MDSC 301 in Winter 2023 and 2024 were eligible to participate in the following research study: “College of Discovery, Creativity, and Innovation: Undergraduate Research initiative – What attributes and activities contribute to quality undergraduate research at UCalgary in the course-based undergraduate research programming (CURE), Global Challenges course, Ready for Research, and the Program for Undergraduate Research Experience (PURE)” (Ethics REB20–2110). Participation was anonymous, voluntary, did not require any additional coursework or research duties, and was not associated with performance, assessment, grades, or merit. Students were informed via email in 2023. In 2024, students were informed via email and in-person presentation of the CURE evaluation project. Both years, information was also sent with the survey. Informed consent was then obtained in writing at the beginning of the questionnaire. The student cohorts from 2023 and 2024 were invited to participate in the study and their answers were collected from February 2024 to April 2024, and from May 2024 to July 2024, respectively.

### CURE implementation

**Fig 1:**
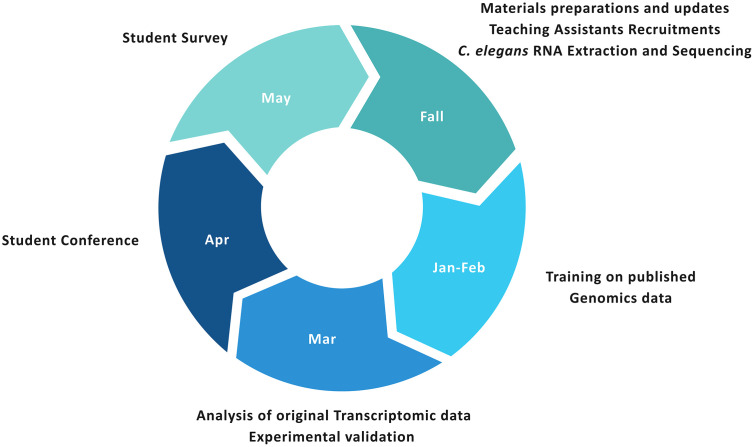
Yearly planification for preparation and implementation of the CURE.

MDSC 301 at UCalgary is scheduled once a year in the Winter semester (Fig 1). All materials are prepared in the Fall, prior to the class. This includes an update of the Lab Book and acquisition of new transcriptomic datasets. TAs are also recruited in the Fall. From January to February, students are guided through the re-analysis of genomics data, as in Maroilley et al. [47,48] to acquire the necessary background knowledge in molecular biology, bioinformatics techniques, and research methods. Then, students analyze their original transcriptomic data in March and present their findings at a conference organized on the last day of the semester in mid-April (Fig 1).

### Schedule

**Fig 2:**
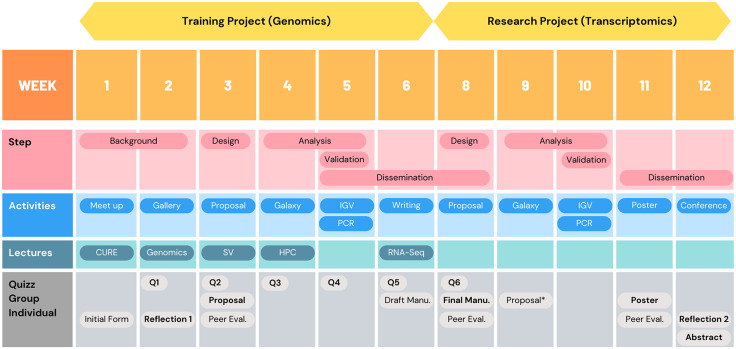
Overview of the semester schedule. At the top of the Gant graph, the distribution of the semester between both projects is illustrated. The first line (yellow) represents the 12 weeks of the semester. The second lane (pink) displays the different steps of the projects that students accomplished at specific times of the semester. The third lane (blue) reports the activities organized in class each week. The fourth lane (green) shows the schedule of lectures during which the students were provided with the necessary background knowledge to fulfill their tasks. The last lane (grey) represents the due date of marked (in bold) and unmarked assignments.

The MDSC 301 CURE is implemented over 12 weeks (Fig 2). In the first 6 weeks, the students work in group on a mock research project: “How to detect SV in short-read whole genome sequencing data?”. The students repeat analyses done on a strain of their choice published in either Maroilley et al., 2021 [47] or 2022 [48], from which genome sequencing data are publicly available. They are guided throughout the pre-processing of the sequencing data, then apply multiple SV callers, and then assess and compare their accuracy.

In the last five weeks of the semester, the students have access to the original transcriptome sequencing data from the same strain they used in the genomics project. In that way, they will study the effects of SVs they detected.

### Materials

The main resource for the students is the Lab Book, made available at the beginning of the semester. It was written by the Instructor and the TAs and inspired by a previous CURE published online [51]. It contains:

- A presentation of the format of the course as a CURE;

- An introduction to the projects (training and research);

- A set of rules and advice to ensure the success of the CURE;

- A detailed schedule, week by week, with every resource necessary to accomplish required activities and assignments.

- Background summaries: To complete the short lectures and active learning activities, several background documents are available in the Lab Book. They summarize introductory information on the main scientific topics necessary for the students to accomplish their projects: *C. elegans* as a model organism, Genome sequencing, Structural variants;

- Templates: for each research-inspired group assignment, detailed templates are available. They describe the expected content of each assignment. For instance, for the manuscript due in Week 8, the template summarizes the main sections of a manuscript (Introduction, Methods, Results, Discussion, and References). Each section is explained and the expected content to obtain the full mark is cataloged. As for the Results section, the template explains that only outcomes for the analysis must be reported in this section, and the expected tables and figures are described;

- Project guidelines: For the Training Project (Genomics), the students are guided at every step via a series of protocols with screenshots, command lines and scripts made available. For the Research project (Transcriptomics), the Lab Book offers the students advice, ideas, and explanations, but the students have the freedom to design their workflow;

- Rubrics for all assignments.

The development of the original Lab Book took about six weeks by the main instructor being helped by some TAs who wrote one or multiple templates or background summaries. Each iteration, the Lab Book is revised and updated based on students’ feedback and observations made by the Instructor and the TAs while implementing the course. Every year, one of the main alterations of the Lab book is related to calendar constraints. Indeed, we found it important to adjust the schedule around the week of the Winter break to avoid any major disruptions to the training project. In addition, during the first iteration, we had a one-hour lecture and a one-hour activity to present R to the students so they could choose to use either Galaxy or R to implement their RNA-Seq analysis. But students with no prior programming experience struggled and reported that it was challenging to grasp anything in such a short time. Ultimately, all groups chose to use Galaxy. In the following years, we decided to remove the Introduction to R but still allow students with prior experience to use it for their project. The last major change was related to the acquisition of a portable PCR machine (miniPCR bio) that allowed the students to try out PCR during the genomics project to validate breakpoints during the second iteration. We added a session of validation of changes in gene expression via RT-PCR with the same equipment during the third iteration.

### Learning Objectives

The course Learning Objectives are multi-faceted as described in Table 1.

**Table 1:** Learning Objectives in MDSC 301.

Type	Learning Objectives
Research	Understand how to design research projects to answer a research question
	Learn about the uncertainness in the research process and the importance of resilience and persistency
	Be aware of and recognize possible biases, errors and misinterpretations
	Identify and use reliable online resources to find relevant information
Dissemination	Summarize a scientific project in the form of an abstract, a poster or a manuscript with relevant figures and tables
	Understand the importance of proper referencing
	Present scientific findings to an audience
Computational Sciences	Be familiar with local and online HPC systems: strengths and weaknesses
	Troubleshoot a bioinformatics tool and workflow
	Choose the right tools based on needs, knowledge and time
Bioinformatics	Understand how biology and bioinformatics are inter-dependent
	Be familiar with the best practices to design/conduct bioinformatics analyses
	Understand the importance of validating findings
	Recognize that Bioinformatics always requires troubleshooting
Collaboration	Recognize the importance of peer support and collaboration
Omics	Understand that one dataset allows multiple approaches
	Be familiar with the processing of genomics and transcriptomics data
	Understand principles and biases associated with sequencing technologies
	Recognize the importance of understanding how the data were produced to anticipate any bias and avoid misinterpretation
Genetics	Be familiar with structural genomic variations
Model organism	Recognize the value of *C. elegans* as a model
	Understand the concept of model organisms
	Interpret results in the model organism context
	Recognize the importance of model organisms for biomedical research
	Avoid over-interpretation of translatability of model organism results
Open Science	Understand the value of Open Science strategy

### The challenge of introducing Applied Bioinformatics to students with different university backgrounds

Originally part of the Bioinformatics program in the Bachelor of Heath Sciences at the Cumming School of Medicine as a second-year mandatory class, the MDSC 301 is opened to students from other programs and other years. On average, over the three years of implementation, only a third of the students were second-year Bioinformatics program. Other students were third-, fourth-, fifth-year students from Biological Sciences, Cell and Molecular Biology, Computer Science, or Biomedical Sciences. Their experiences in Bioinformatics were either none, a block-week introductory class, a few classes, or summer internships in research laboratories working on projects involving Bioinformatics.

To address the challenge of conducting a research project with a Bioinformatics approach that would be accessible and engaging for all students, several measures were implemented:

1. At the beginning of the semester, students were asked to complete a survey answering questions to evaluate their experience in Bioinformatics, Genetics, and research (See S1 File). This allowed the instructor to adjust the curriculum to the current cohort of students.
2. Provision of a Lab Book with tutorials for every step of the Training Project (Genomics) and guidelines for the Research Projects (Transcriptomics) (https://github.com/MTG-Lab/MDSC301).
3. The core of the Training Project (Genomics) was adapted to the free online platform Galaxy [52] (Europe), allowing anyone with no experience in UNIX systems, command-line or coding to run Bioinformatics analyses. The most popular tools have been made adapted to Galaxy for easy utilization.
4. As an option for students majoring or minoring in Bioinformatics, or with experience or interest in using command-line, scripts and UNIX system, we provided access to a local High-Performance Computing system (TALC, University of Calgary). As such, when other students ran SV callers on Galaxy, volunteers would run similar callers pre-installed on the cluster. A SLURM script template was provided to facilitate running a job and guidance during class hours. The instructor and TAs were available during office hours and via email to students. To avoid students feeling pressured in choosing one of the options, grades were not affected by the system used.
5. Teams were built based on answers received via the initial survey (S1 File). In brief, we collected names for students they would trust to work with, but also their experience in research, Bioinformatics and Genetics, and their self-assessed skills in writing and presenting. As such, we built teams of four students as follows: i) accommodating group requests; ii) one member of each group should have prior Bioinformatics/research experience; iii) one member of each group should have good writing skills.
6. Hiring teaching assistants with a Bioinformatics background to ensure mentorship to students who need it.

### Fostering (smooth) collaboration

Bioinformatics suffers from stereotypes, often leaving students thinking that Bioinformaticians or researchers in Bioinformatics work alone. However, collaboration is essential in Bioinformatics, as it is in research, but even more so as the field is rapidly evolving and knowledge is not always recorded in books or published as articles. In addition, collaboration is a transferable skill, valuable in any path students will be joining after their degree.

To foster as much collaboration as possible in the class, most of the research-like assignments were to be done in groups. Therefore, MDSC 301 implemented a Team Based Learning approach: the groups are formed by the instructor, yet the team members evaluate each others contribution. This approach was announced to the students in the first class to give them time to raise any concerns and get familiar with the concept before any group assignments. Additional information, advice, and good practices were available to the students in writing in the Lab Book. In brief, the main research assignments were to be done in groups: the Training Project (Genomics) proposal (10% of the grade), the Training Project (Genomics) manuscript (20% of the grade), and the Research Project (Transcriptomics) poster (20%).

To give the groups the opportunity to get to know each other before group assignments, we formed the teams as early as possible in the semester (second week), and we planned active learning activities in groups to get team members to work together without the pressure of group assignments for a few weeks. In addition, group work can only be successful with efficient organization including task allocation and milestones. But such planning requires understanding what precisely the assignment unfolds. We then decided to guide the students in their project management of the Training Project (Genomics) to avoid setbacks and pitfalls that students would encounter just due to bad planning. As such, we gave them an example of how to divide the tasks in their group to allow each student to work in parallel on similar but independent tasks. This project management allowed mutual assistance in groups but also among groups but still gave each student responsibility for an essential part of the project.

We used individual feedback to give an opportunity to each student to report any issue in their group and ask for help individually. Feedback was collected at multiple time points during the semester through different formats:

1. Peer-evaluation: With each group assignment due, students were due for an individual peer-evaluation. They would assess the engagement, reliability, and efficiency of each of their teammates and themselves. The peer-evaluation would affect a student grade only if on average a student would receive a peer-evaluation lower than 10/20. Otherwise, it was used to spot any minor issue in the group that would have arisen.
2. Reports: Reports are individual and questions were deliberately oriented to make students reflect on their performance as an individual and as a teammate. Some questions were also designed to gather information about group organization while giving advice to the students about how to improve group work (see S1 File).
3. Anonymous feedback: As not every student would feel comfortable speaking up, we also made available throughout the semester a link to an online survey that allowed anonymous feedback.
4. Daily availability: the instructor and the teaching assistants were always attending classes, offering office hours, and responding in a timely manner to emails to create and maintain trust for students that they feel supported and welcomed to reach out.

### Engaging students in an active learning journey

For students to acquire the necessary knowledge to conduct the Research Project (Transcriptomics), we implemented various learning activities to engage the students in an active learning process, but also as an attempt to satisfy all learning styles.

First, all lectures were less than 30 minutes, aiming to deliver the main information necessary to the execution of a part of the project. Slides were made available to the students and were the main resources for quizzes. Then, summaries of basic knowledge were included in the Lab Book, giving the students the opportunity to always get back to this resource. If students wanted to come prepared in class or to explore more deeply a subject, for each session, optional readings under various formats were listed in the Lab Book (videos, free textbooks, free research or review articles). Finally, in any literature search that was necessary, a short list of meaningful articles was always proposed to offer to guide the students in their search while providing fundamental information.

Several active learning activities were implemented:

1. “Meet up with the worms and the team”: To introduce the students to the concept of research with model organism (here *C. elegans*, a 1 mm-long roundworm), we organized a visit of a research group in the classroom. For one hour, students observed worms under the microscope and discussed with graduate students and postdocs who work with *C. elegans* daily. In addition, we invited graduate students and postdocs working essentially on computational Biology techniques. The meet-up was guided via an individual written assignment of six questions, designed to make students ask relevant questions to the scientists regarding research, Bioinformatics and *C. elegans* (see S1 File).
2. Gallery Walk: Previous research showed that active learning activities such as Gallery Walks promote discussion and facilitates knowledge acquisition [53–55]. Prior to the session, students were asked to read pre-selected articles (four per group, so one per student) related to different subjects they would have to discuss during the activity (subjects available in the Lab Book). In class, students were asked to write relevant information to different themes displayed on white boards (e.g., Sequencing technologies” or “Biases in RNA-Seq”). Every ten minutes, each group walked towards the next board and built upon what was already written by previous groups. This activity was implemented twice: once for genomics and once for transcriptomics fundamentals.
3. As part of the research projects, the students tried to validate some of their findings experimentally. We acquired the miniPCR Bio kit, the miniPCR thermocycler and the blueGel electrophoresis system, a portable technology allowing to perform PCR in any environment, including a classroom (https://www.minipcr.com/). To fit in with the 1.15-hour session, students were instructed to design primers; however, the primers were designed and ordered by the instructor ahead of time. DNA or RNA extraction, PCR mix and gel preparation were performed by the graduate assistants before the class. Students loaded the gels with their samples. Gel was run in the classroom in front of the students, who, thanks to the blueGel electrophoresis system, could follow in real time the progression of the experiment. An example of gel is available in S1 File.
4. Mini-symposium: Toward the end of the semester, students were engaged in a demanding research project as they analyzed the transcriptomics data to study the effect of SVs on gene expression. To allow them to fully focus on their research, we did not impose a final exam. In term of final assignment instead, we required the design of a poster. To promote engagement and genuine motivation, we organized a one-hour mini-symposium like Summer Research symposiums on the last session of the course. The event was advertised to gather students and researchers at the event and give a chance to the students to present their work and findings to a varied audience and discuss their findings. We chose not to mark their participation at the conference so that the students could focus their efforts on presenting their findings and confronting their ideas with peers, as it happens in professional conference, without the pressure of following a rubric to get a good mark.

### Supporting students in research-like assignments

To allow students to experience the research process from designing a project to disseminating results and conclusions, we planned research-like assignments: proposal, manuscript, abstract, and poster. However, such assignments can be overwhelming for undergraduate students as each format has a set of rules and best practices that scientists usually learn-by-doing over several years in a lab. In the context of a CURE, it was also important to make sure that the assignments would not take over the bioinformatics analysis that students were conducting. Then, to allow easy time management for students, and guide them into the production of research-like assignments while learning the best practices, we created detailed templates and guidelines made available in the Lab Book (see Lab Book).

In addition, we planned a major part of each session in class to be a time for students to work on their projects and assignments. As such, any question or issue arising could be addressed immediately by the instructor, the teaching assistants or peers, avoiding any delay in the completion of the assignments. During work sessions in the class, the teaching team talked to each group to ask for updates and offer help. We found this approach important to reach students reluctant to ask questions. It was also critical in noticing teams that were encountering delays and providing personalized support to help them to be back on track. Work sessions also favored collaboration inter-teams, even more encouraged by the instructors: when some teams would struggle obtaining a file for technical reasons, “colleagues” working on the same sample were asked to share their file allowing everyone to pursue their analysis.

For each research-like assignment, early deadlines were set to allow students to pre-submit a draft of their assignment to receive feedback before finalization and final submission. The feedback would highlight any missing requirement, advice on the format and the content, review the quality of the writing for the available sections, and suggest techniques to improve/complete the draft and any remaining analysis in a timely manner.

Finally, the rubrics were made available and reflected the marking system. We graded the research-like assignment based on their alignment with the requirements described in the template – “was all required information presented in the appropriate format?” The grade was not affected by the scientific findings, as an attempt to allow students to conduct their analysis without the pressure of finding the “right answer” as in usual assignments. Here, we favored the intellectual process behind the analyses and the dissemination of the results over the accuracy of the results. We also encouraged and guided the students in explaining, justifying, and reflecting on possible biases and limitations in their chosen methods as is done in scientific publications.

### Helping students evaluate their learning

As previously described, the MDSC 301 CURE allows students to acquire, develop, or improve bioinformatics core competencies, and transferable skills. As a CURE can be confusing in terms of how it will beneficiate students who do not wish to pursue research and/or in the specific field of research in which the project fits, we implemented resources similar to meta-cognitive assessments to favor students self-assessment at the beginning and the end of the semester, encouraging them in reflecting on their journey and their learnings.

First, the Initial Survey (see S1 File) asked several questions to each student regarding their outlook of Bioinformatics and research, current questions, and expectations for the course. Then following the “Meet up with the worms and the team” activity, previously described, students were required to complete their first individual assignment (Reflection 1, see S1 File). The format was a series of questions regarding the observation of worms and the discussions with model organism biologists and computational biologists. The questions were encouraging self-reflection regarding how this experience changed, or did not change, their understanding of research, Bioinformatics, and model organisms. The final individual assignment, called Reflection Assignment 2 (see S1 File) was designed to mirror the first two self-reflection activities by asking similar questions to the students at the end of the semester and allowing them to reflect on their growth in terms of scientific knowledge, transferable skills, but also as a person.

### Sample collection and data acquisition

At each cycle of the course, three different *C. elegans* balancer strains were analyzed (Table 2)

**Table 2:** Bioinformatics core competencies.

Bioinformatics Core Competency	Definition	Details
**S1 (Role)**	Understand the role of computation and data mining in hypothesis-driven processes within the life sciences	Bioinformatics analysis design (pipeline) to answer a biological question
**S2 (Concepts)**	Understand computational concepts used in bioinformatics	Pipeline design, tools, file formats (Fastq, Fasta, Bam, VCF)
**S5 (Tools genomic)**	Be able to use bioinformatics tools to analyze genomic data	FastQC, Trimmomatic, MarkDuplicates, BWA, SV callers, IGV
**S7 (Tools expression)**	Be able to use bioinformatics tools to analyze gene expression data	Cutadapt, STAR, FeatureCounts, HTSeq-count, Limma, DESeq2, OUTRIDER, FRASER
**S13 (Scripting)**	Know how to write short computer programs as part of the scientific discovery process	(OPTIONAL) Bash, R, python
**S14 (Software)**	Be able to use software packages to manipulate and analyze bioinformatics data	See S5 and S7, Spreadsheets
**S15 (Computational environment)**	Operate in a variety of computational environments to manipulate and analyze bioinformatics data	Web-based, (OPTIONAL) Unix/Linux command Line

For the Training project, genomics data were collected from Maroilley et al., 2021 and 2023. For iteration of the CURE, N2 and Hawaiian (CB4856) strains were used as a control, and three balancer strains were made available to the students:

- 2023: BC986, BC4586, and VC109
- 2024: CZ1072, MT690, and SS746
- 2025: BC1217, GE2722, and RW6002

For the Research project, total RNA was extracted from a pellet of about 1,000 worms with a Trizol-chloroform based RNA extraction protocol followed by in-column DNAse treatment ZYMO Research RNA Clean & Concentrator kit. (ID: R1013). Purified RNA was eluted in DNAse-RNAse free water provided by the manufacturer (ZYMO Research). RNAs were depleted from ribosomal RNAs. mRNAs were then sequenced at the Centre for Applied Genomic Next Generation Sequencing Facility (SickKids, Toronto, Canada) with Illumina NovaSeq 6000. For each student cohort, N2 was used as a control.

### Genomic data analysis

The genomic data, 150-bp Illumina short reads, were derived for the genome of *C. elegans* balancers stored in fastq files. Raw data were preprocessed as in Maroilley et al., 2021 and 2023, on the online HPC system Galaxy (Galaxy Europe at https://usegalaxy.eu). Each student created a free account on Galaxy Europe (https://usegalaxy.eu/). Data were uploaded in “History” by the instructor and later shared with the students. The quality control of the data was checked with FastQC [56]. Trimmomatic [57] was then ran on the raw data to trim reads from bad quality bases, discard unpaired reads, and remove adapters. Duplicated reads were marked with MarkDuplicates (Picard) [58]. Reads were then aligned to the *C. elegans* reference genome (ce11) using BWA-MEM [59]. Quality of the alignment was evaluated using FastQC [56]. SVs were called using Delly [60], Lumpy [61] and Basil [62], available on Galaxy. SVs were also called with Manta [63], SeekSV [64], GRIDSS [65], and TIDDIT [66], ran on a local HPC system (TALC, University of Calgary). Calls were filtered based on quality scores of each caller, overlapping between callers, and visual inspection of breakpoints using Integrative Genomic Viewer (IGV) [67].

### Transcriptomic data analysis

The transcriptomic data, 150-bp Illumina short reads, were derived from the *C. elegans* balancers stored in fastq files. Transcriptomic data pre-processing was implemented on Galaxy Europe. Quality of the raw data was assessed with FastQC [56]. Reads were trimmed with Trimmomatic [57]. Adapters were removed with either Trimmomatic [57] or Cutadapt [68]. Reads were aligned to the reference genome with STAR [69] or HISAT2 [70]. Manual analysis was performed on IGV [67]. Read counts were obtained using featureCounts [71] or HTSeq-count [72]. Differential expression analysis was made using limma [73], allowing the absence of replicate in the balancer group, or DESeq2 [74], either on Galaxy Europe (https://usegalaxy.eu/) or R (https://www.r-project.org/).

### Experimental Validation

Breakpoints were validated by PCR using DNA. Gene expression levels were studied via RT-PCR using cDNA. The PCR reagents were mixed as follows: 15 µL or water, 4 µL of 5X EZ PCR Master Mix, 0.4 µL of 10 µM primers, and 0.6 µL of worm lysate. The PCR thermocycler was run as follows: initial denaturation 95˚C for 3 minutes, denaturation 95˚C for 30 seconds, primer annealing 54˚C for 30 seconds, extension 72˚C for 45 seconds, and final extension 72˚C for 5 minutes. Denaturation-annealing-extension was repeated 35 times. Gel electrophoresis performed for 30 minutes at 100V using a 2% agarose gel loaded with 10 µL of PCR mix (1:5 dye:sample).

## Results

### The MDSC 301 CURE had all characteristics of a CURE

CUREs are defined by five main characteristics that our course fulfilled [23]:

1. Scientific process: The students were going through the scientific process twice over the course of this CURE, once during the Training project (Genomics), and once during the Research project (Transcriptomics) with more guidance the first time. Each time, students had access to a short list of research and review papers in which they could find relevant information to design a project addressing a specific question (“How to detect SV in genome sequencing data?” and “Which effect can SV have on gene expression?”).
2. Discovery: The data used during the Research project (Transcriptomics) were original, obtained by a research laboratory and not yet analyzed. Any result obtained by the students was in this way an authentic discovery.
3. Relevance: SVs and CGRs are known to change the structure of large genomic regions, and it is to be expected that this would have consequences on downstream phenotypes (RNA, protein, and visible phenotypes). However, there are currently no standard ways to interpret the impact of an SV computationally and SVs often remain ambiguous in genetics and genomics studies. By exploring the effect of many various SVs, we can pave the way to developing prediction tools.
4. Collaboration: Research projects have been designed in a way so that goals can be achieved via the collaboration of at least four students. In addition, we encouraged collaboration between teams.
5. Iteration: During the Training project (Genomics), students were following a protocol provided in the Lab Book. By doing it themselves, students faced the day-to-day challenges in Bioinformatics and got to re-try and troubleshoot (learning by failure and retrying). For the Research project (Transcriptomics), students designed their analysis, involving making choices and having to adjust them (revising thinking).

### A design addressing core competencies and translational skills

The Network for Integrating Bioinformatics into Life Sciences Education (NIBLSE) has published a list of 15 bioinformatics core competencies [75]. Table 2 summarizes the seven Bioinformatics core competencies students are familiar with in the MDSC 301 CURE. S13 and part of S15 (Table 2) are based on activities in the Training Project (Genomics) made optional for students with previous bioinformatics or computational sciences experience, or particular interest in the usage of command-line, scripts, and UNIX systems (see “The challenge of introducing applied bioinformatics to students with different university backgrounds” section for more details).

As students experienced research, they developed or reinforced interdisciplinary and transferable skills. Table 3 summarizes the transferable skills supported by the MDSC 301 CURE.

**Table 3:** Transferable skills.

Skills	Definition
**Collaboration**	Work respectfully and efficiently with others, plan a collaborative project with milestones.
**Communication**	Learn and share information by presenting orally and in writing, listening, and interacting with others.
**Creativity**	Elaborate on new approaches.
**Critical thinking**	Actively and skillfully conceptualize a solution and apply a method. Analyze, synthesize, and evaluate results/information to draw a reasonable conclusion.
**Digital skills**	Use digital technologies (computers, high performance computing system, online resources).
**Literacy**	Find, understand, and use information presented through words.
**Numeracy**	Use mathematical information.
**Problem Solving**	Identify an issue, know how to look for (online, literature, peer support), find, implement and assess a mitigation.
**Mentoring**	Peer-mentoring based on individual strengths and university background.

### Impact of MDCS 301 CURE on student experiences

**Fig 3:**
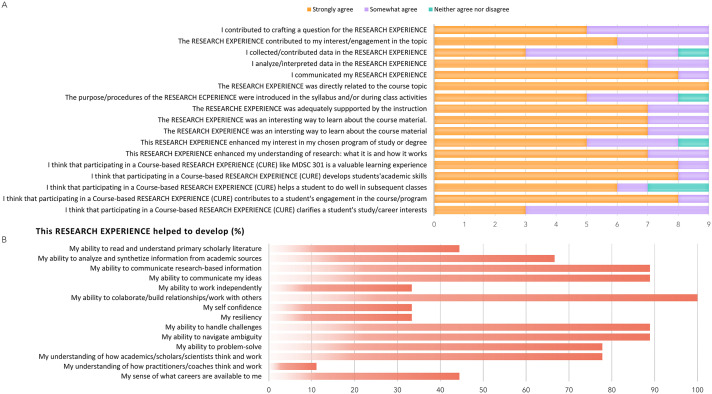
Survey results. A) Bar plots summarizing the answers of the nine participants to the survey. Students who gave their consent to participate in the study were administered an online form including a series of 17 questions for which they could answer “Strongly agree”, “Somewhat agree”, “Neither agree nor disagree”, “Somewhat disagree”, or “Strongly disagree”. As none of the nine participants ever answered, “Somewhat disagree” or “Strongly disagree”, we removed these options from the Fig 3 to improve readability of the graph. Questions were ordered on the graph as in the survey. B) Histograms (%) of the answers selected by the nine participants to the answer “This Research Experience helped to develop…”. Participants could select none, one or multiple answers. If the histogram reaches 50%, it means that 50% of the participants pick that answer.

As part of the study REB20–2110, a post-course survey was conducted on voluntary basis for the 2023 and 2024 cohorts (see Methods). Overall, nine (4 students in 2023, 5 in 2024) participated (14%). In sum, students who participated in the survey reported that the MDSC 301 CURE had positive effects on their learning. Survey’s participants also felt that the CURE had a positive effect on their engagement in their program and clarified their interests for future studies or career (Fig 3, Table 4).

**Table 4:** Open statements.

Open statement	Sample responses
As a result of this RESEARCH EXPERIENCE, I…	Learned more about Bioinformatics and what encompasses as a field.
	Have a better understanding of the research process and how research is conducted in the real world.
	Have much more thorough understanding of research approaches in Bioinformatics, specifically in RNA sequencing.
	Acquired a deeper understanding of the importance of team collaboration in scientific research, fostering teamwork, negotiating, and compromise while working towards a common goal of producing high quality research output.
Participating in a RESEARCH EXPERIENCE meant…	I was able to work with my peers and collaborate to produce a final project.
	That I got to actually apply what had been taught and not simply absorb theoretical information.
	I got an opportunity to work in a team to complete rigorous research projects in an area of personal interest.
	Developing essential skills such as critical thinking, problem-solving, and effective communication within a collaborative team setting, fostering a holistic approach to learning beyond traditional coursework.
This RESEARCH EXPERIENCE helped develop…	Skills in creating a pipeline and analysis related to it.
	Skills such as making a poster to present relevant findings.
	My passion for Bioinformatics and for research as a whole.
	My ability to perform RNA sequencing analyses.
	My presentation skills significantly, particularly through the creation and delivery of our poster presentation during the mini-symposium. It honed my ability to distill complex information into a concise and visually engaging format, as well as to effectively communicate our findings for a diverse audience, It offered a safe place to refine my skills in a setting that mirrored professional conferences, ensuring I entered future endeavors equipped with confidence and proficiency in scientific communication.

### An example of scientific discovery: *eT1* disrupts more than the expected *unc-36*

*eT1(III;V)* is a reciprocal translocation used to balance the right end of chromosome III and the left end of chromosome V in *C. elegans*. Breakpoints are III: 8,200,764 and V: 8,930,675. *eT1(III;V)* is easily traceable in a colony as its presence produces an uncoordinated phenotype. It was suspected that this phenotype was due to the disruption of the *unc-36* gene, situated at the chromosome V breakpoint. However, little was known regarding the potential effect of the chromosome III breakpoint, as it is located in an intergenic region.

We sequenced the transcriptome (bulk RNA) of the BC986 strain, carrying the *eT1(III;V)* reciprocal translocation. Visual observation of the RNA-seq alignment using the IGV performed by the students helped further understand the effect of *eT1*.

**Fig 4:**
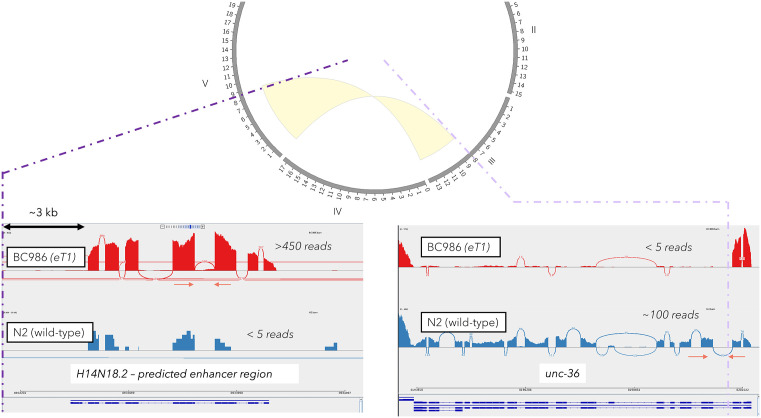
*eT1* in the BC986 strain, and effect on its transcriptome at both breakpoints. On top, the Circos plot shows the reciprocated inverted translocation with both breakpoints on chromosome III and V. At the bottom left, Sashimi plot produced with IGV showing the read coverage of the exons of the H14N18.2 gene in BC986 and N2 gene. At the bottom right, Sashimi plot showing the read coverage of the unc-36 gene in BC986 and N2 strains.

At the chromosome III breakpoint, the transcriptome data showed a different expression of the *unc-36* gene, when compared to control: in BC986 only exons 1 and 2 were well covered by reads, while the rest of the *unc-36* presented almost no coverage. In comparison, in N2 (control), the level of coverage was stable throughout the whole transcript. This suggested a truncation of the transcript, which aligned with the location of the breakpoint, just left of exon 2 (Fig 4).

At the time of the analysis, the chromosome V breakpoint did not overlap any known annotated genetic element (coding or non-coding). However, a visual analysis of the RNA-seq alignment showed a notable variation in the coverage of *H14N18.2*, predicted enhancer region, located at about 3 kb from the *eT1* breakpoint (Fig 4, left panel). The pattern of coverage showed clearly distinctive exons that were barely covered on the N2 control. This suggested that the *eT1* breakpoint induced an expression of *H14N18.2*, usually not expressed in N2. As *H14N18.2* remained understudied, its expression in an *eT1* balancer-containing strain gives material for further investigation of this genetic element in the *C. elegans* genome. We used an RT-PCR to validate the over-expression of *H14N18.2* in BC986 when compared to N2 (S1 File).

## Discussion

The fields of Bioinformatics, Computational Biology, and Data Science are exponentially expanding with the advent of high-throughput technologies and Artificial Intelligence. Multi-disciplinary training is necessary, but the offer remains limited and often implemented in post-secondary institutions for graduate students. This impacts directly the limited interest of students in any field related to Bioinformatics, which suffers from a stereotype of a narrow, impersonal, and unsuited field for those who wish to work on a human level, discouraging students from pursuing these paths. Implementing additional introductory courses in Bioinformatics and programs at undergraduate levels would foster interest in students, as they would have a better understanding of what Bioinformatics involves. But it has been shown that in the forms of lectures with theorical content, introductory Computer Science courses play a big role in discouraging students from majoring in computer science, especially women. To foster student engagement in Bioinformatics-related career, it is then of the utmost importance to improve introductory curriculum, tailoring how we teach Bioinformatics by “marketing” the courses [75]. Here, we designed and implemented a CURE as an introduction to Bioinformatics, in the form of a research project with applied Bioinformatics techniques on omics data. In brief, we combined multiple approaches to engage all students regardless of learning-types. We combined authentic research projects, original data, active learning activities, research-like assignments and open-to-public mini-symposiums.

A post-course survey indicated that students’ interest and understanding in the field have improved. However, we acknowledge that the low participation in the survey (14% of students enrolled in Winters 2023 and 2024), is a limitation to our study and may not be applicable to all students [76]. Furthermore, considering that the survey was anonymous, we did not ask for any identifiable information such as ethnicity, major, or experiences. Therefore, we cannot consider for disparities of students’ experiences based on their background. In addition, survey’s participation was voluntary, and it was released after the grades were submitted, to avoid perception that the survey could affect students’ grades. Considering this, we were only left with a possibility to administer survey via online form, which could have affected the participation rate due to digital survey fatigue [77,78]. This may explain a lower response rate as students often rapidly move on after the end of the Winter semester to new endeavors and might not be motivated to look back or may not even regularly check their student email boxes. However, the data we gathered still provides valuable insights. Future work would benefit from pre- and post-surveys to assess the growth of the students throughout the course [79]. Also, surveys could be part of the course but with participation in the research study still optional. In addition, reflection assignments could be included in the assessment strategy with the appropriate approval from IRB and consent from the students.

Undergraduate Computer Science programs remain remarkably un-diverse, with women only earning 18% of computer science bachelor’s degrees in the United States for instance. Women and other minority groups are also dismal in the computer science professions, with fewer female authors, in sex or gender, on research papers, as compared to biology in general (~20–30%) [80] and a persistent under-representation of minorities in the field [81,82]. With the implementation of a CURE, we aimed to support students from underrepresented groups and to create an inclusive learning environment, where all students feel that their differences are valued and respected, have equitable access to learning and other educational opportunities, and are supported to learn to their full potential. As such, suggested materials were exclusively focused on accessible, free resources, such as university computers, and the analyses that utilize accessible resources such as an online free platform (Galaxy) or a computing system available to students at UCalgary.

Here, we intended to disseminate our curriculum, and all materials and strategies developed to allow implementation of this CURE in other programs or facilitate the design of other CUREs by providing ideas, support and resources. Indeed, designing a CURE from the start can be intimidating and instructors might be reluctant due to the charge of work it might represent. Therefore, we choose to share with the community our materials to reduce this burden and support further development of new CUREs in this field. Our CURE was inspired by Villa-Cuesta & Hobbie [51] in its form but developed around an original research project developed in the Dr. Tarailo-Graovac laboratory and based on instructor expertise. By providing all resources, and in the era of Open Science with most datasets being publicly available, even instructors with not the exact skillset for this project could implement this course at least partially.

Any data science research project is designed around three essential elements: a research question, a dataset, and a method of analysis. To design a successful CURE as an Introduction to Bioinformatics, the main caveat is to think that students need independence to conduct their research. On the contrary, undergraduate students in a class, just as summer students in a laboratory, need a reliable support system and a framework for successfully conducting their research efficiently. As such, it is important to design a project by providing the students with at least two of the three essentials (question, method, data). In MDSC 301, we chose to have students working on the same research question (effect of SV on gene expression), and to provide them with the datasets. They could then innovate on the methods to analyze the data to answer the research question. One could choose to let the students find their own research question but provide a specific dataset or a repository (e.g., TAGC) and a method (e.g., a differential expression analysis). Alternatively, students could be provided with a method to apply a specific question, but they would have to find their own dataset. Our design was based on research in which the instructor was involved. As such, CURE provides the perfect opportunity to combine both research and teaching activities, both often required from academics.

## Supporting information

S1 File Supporting information. (DOCX)
