# Supplementary material for: A course-undergraduate research experience (CURE) to explore the effect of structural variants on gene expression in C. elegans balancers
Source: PLoS One. 2026 Aug 11;21(8):e0342806. doi: 10.1371/journal.pone.0342806 (PMC13460597; doi:10.1371/journal.pone.0342806)
Supplement: Supplementary file 1 [file pone.0342806.s002.pdf]

COMMENTS TO AUTHORS

Manuscript PONE-D-25-66689

A Course-Undergraduate Research Experience (CURE)
to explore the effect of structural variants on gene expression in C. elegans balancers

Major Comments

The following issues are considered essential for the manuscript to meet publication standards. All must be addressed in revision.

Educational outcome claims are not supported by the survey data

[Lines 499–503; Abstract lines 45–48]

The central educational claim that the CURE 'favored students' understanding of the Bioinformatics field and fostered their research interest' is not supported by the available evidence. Only **9 students** (4 from the 2023 cohort, 5 from 2024) participated in the post-course survey, representing a 13% response rate from approximately 65 eligible students. The entire 2025 cohort (44 students) is excluded from the analysis.

This is not merely a statistical power limitation; it is a fundamental validity problem. Voluntary post-course survey participation is systematically biased towards students who were more engaged and had positive experiences. The students most likely to be dissatisfied, anxious, or disengaged, the population that matters most for evaluating inclusive design claims, are absent from the data. Any conclusions drawn from 9 self-selected respondents cannot be generalized to the course's impact.

The abstract further states that the course 'favored students' understanding and fostered their research interest' without any qualification of the sample size. It is likely that readers unfamiliar with the Methods will assume these claims are based on representative data.

Suggestions for revision -

- Reframe all educational outcome language as preliminary and exploratory throughout the abstract, results, and discussion. Replace causal and generalizing language ('showed that,' 'favored,' 'fostered') with appropriately hedged terms ('suggested,' 'was consistent with,' 'reported by respondents').
- Conduct and report a matched pre-post comparison using the Initial Survey (already administered) as a baseline. Even with n=9, within-subject comparisons are more informative than post-hoc self-reports alone.
- Consider collecting survey data from the 2025 cohort to increase the sample, or explicitly state why this was not possible and when it will be reported.
- Add a dedicated limitations paragraph in the Discussion addressing self-selection bias and the constraints of the current sample size.

No control group and no pre-post knowledge measurement

Without a comparison condition (e.g., students in a traditional bioinformatics lecture course), it is not possible to attribute observed positive perceptions to the CURE format specifically. Similarly, without matched pre-course and post-course knowledge assessments, the survey data will not be able to substantially demonstrate learning gains; it can only demonstrate satisfaction, which is a distinct and weaker metric.

The Initial Survey and the Post-course Survey differ substantially in format: the Initial Survey uses open-ended, yes/no, and multiple-choice formats, while the Post-course Survey uses Likert scales and open statements. This limits the types of comparisons possible, but there are meaningful matches across three tiers of quality.

Tier 1: Strong Direct Matches (same construct, quantifiable pre-post comparison possible)

Initial Survey	Post-course Survey	Construct
Q8: "Are you comfortable working in groups?"	Q11 sub-item: "My ability to collaborate/build relationships/work with others"	Collaborative ability / group work comfort
Q9: "Are you comfortable talking in public during presentations?"	Q11 sub-item: "My ability to communicate my ideas"	Oral communication confidence
Q10: "Are you comfortable writing essays in English?"	Q11 sub-item: "My ability to communicate research-based information"	Written communication confidence

These three pairs are the most tractable for a proper pre-post comparison. The Initial Survey yes/no comfort ratings can be treated as an ordinal baseline and compared against the Likert-scale post-course self-assessments. Even though the scales differ, the direction of change is interpretable: a student who answered "No" at baseline and rates high improvement post-course provides meaningful signal. With n=9, a matched within-subject analysis (e.g., Wilcoxon signed-rank or descriptive comparison) on these items would be feasible and would substantially strengthen the manuscript's educational claims.

Tier 2: Moderate Conceptual Matches (thematic comparison possible, not directly quantifiable)

Initial Survey	Post-course Survey	Construct	Limitation
Q13: "Three things you expect from MDSC 301"	Q19: "As a result of this RESEARCH EXPERIENCE, I..."	Expectations vs. actual outcomes	Both open-ended; needs thematic coding
Q14: "Two things you already know about Bioinformatics"	Q11 sub-item: "My understanding of how academics/scholars/scientists think and work" + Q13: "My understanding of research: what it is and how it works"	Prior vs. post-course knowledge of bioinformatics	Initial is open-ended; post is Likert

Q15: "One thing you are wondering about Bioinformatics"	Q3: "The RESEARCH EXPERIENCE contributed to my interest/engagement in the topic" + Q12: "My interest in my chosen program of study or degree"	Curiosity / interest trajectory	Format mismatch; needs qualitative coding
Q11 teammate self-description (responsibility, deadlines sub-items)	Q11 sub-items: "My resiliency," "My ability to handle challenges," "My ability to navigate ambiguity," "My ability to problem-solve"	Research resilience and self-efficacy	Initial is categorical (pick all that apply); post is Likert

These pairs allow thematic pre-post narratives but not clean statistical comparisons. They could be presented as illustrative case comparisons per student respondent, which would be honest and informative given the small sample size.

Tier 3: Weak or Indirect Links (background context only, not true pre-post pairs)

Initial Survey	Post-course Survey	Notes
Q4–Q5: Prior bioinformatics classes and experience	Q11 sub-items: ability to analyze/synthesize, problem-solve	Baseline experience may moderate outcome, but these are not measuring the same thing
Q6–Q7: Prior genetics/model organism experience	Q7: "The RESEARCH EXPERIENCE was directly related to the course topic"	Prior knowledge may affect perceived relevance, but these are not matched items

These are useful as covariates or moderators but should not be framed as pre-post matched pairs.

Reference 16 (Wolkow et al., 2019), cited in this manuscript, specifically found that CUREs produce sustainable knowledge gains but only transient perception gains. This is a directly relevant finding that the authors should engage with, as it raises the question of whether the positive survey responses reported here represent genuine durable change or a short-term enthusiasm effect.

- If a control group is not feasible given the institutional context, stating this explicitly and discussing it as a limitation will help the readers.
- Use the Initial Survey data as a pre-course baseline where matched items exist, and report this within-subject comparison.
- Consider engaging directly with the Wolkow et al. (2019) finding in the Discussion and address whether the current survey design can distinguish durable knowledge gains from transient perception gains.

The Introduction lacks a clear logical structure and is missing a critical bridging argument

[Lines 81–114]

The Introduction shifts across four distinct topics in rapid succession, (a) CURE pedagogy, (b) the history of bioinformatics, (c) SV/CGR genomics biology, and (d) *in silico* prediction tools, before arriving at MDSC 301. The transition from line 102 to line 103 is particularly abrupt: the paragraph ending at line

102 closes on the inadequacy of in silico SV/CGR prediction tools, and the paragraph at line 103 immediately pivots to University of Calgary institutional programs. The conceptual bridge is entirely absent.

The paragraph on SVs and CGRs (lines 81–102) is scientifically interesting but its relevance to the CURE is never made explicit in the Introduction. Why does the inadequacy of *in silico* SV prediction tools make an undergraduate research experience the appropriate response? There is a strong argument that can be made - student-led transcriptomic analyses of balancer strains can help populate the evidence base that prediction tools need.

- Adding a bridging sentence or short paragraph between lines 102 and 103 makes the logical connection explicit. The sentence below illustrates the type of bridge needed:

  Suggested revision: *Undergraduate research projects analyzing transcriptomic effects of known SVs in model organisms represent a scalable approach to generating the empirical evidence that in silico prediction tools currently lack. Course-based undergraduate research experiences (CUREs) offer an especially productive framework for generating such data at scale.*
- Restructure the Introduction around two converging threads: (1) the need for bioinformatics CUREs, and (2) the scientific gap that SV transcriptomics in *C. elegans* can address. These threads should converge on MDSC 301 as the solution to both.

Moderate Comments

The following concerns do not prevent publication on their own but would strengthen the manuscript and should be addressed in revision.

No statistical analysis is reported for the key scientific finding

[Lines 507–531; Figure 4]

The paper's scientific contribution that the eT1(III;V) translocation induces aberrant expression of H14N18.2, a predicted enhancer region typically unexpressed in wild-type N2 is presented without any quantitative evidence. The entire finding rests on visual inspection of IGV read coverage tracks (Figure 4). There is no explicit mention of fold-change, normalized read counts, p-values, or adjusted p-values reported for H14N18.2 or for the *unc-36* truncation finding.

This is an obvious omission. The differential expression tools described in the Methods section, specifically limma, which the authors note accommodates the absence of replicates in the balancer group, are precisely the tools that should have been applied to generate statistical results for these findings. Their absence means that a result described as a 'scientific discovery' in the sub-heading has no quantitative basis in the results section of the paper.

Additionally, the Methods section describes PCR validation of gene expression (lines 252–260), but no PCR results are reported for H14N18.2 specifically. It is unclear whether this validation was performed for this gene.

- Report differential expression analysis results (at minimum: normalized read counts, fold-change, and p-value or a relevant equivalent) for both unc-36 and H14N18.2 in BC986 vs. N2.
- Report PCR validation results for H14N18.2 if the experiment was performed, or explicitly state that it was not and explain why.
- Consider presenting comparable data from at least one additional strain (from the 2024 cohort) to demonstrate that the course generates broader scientific value beyond a single case study.

The supplementary document labels the image simply as *"An example of PCR gels (MDSC 301 2024)."* It is placed after the peer evaluation rubric and before the post-course survey. It is presented as a general illustration of student PCR work during the 2024 course iteration.

Claims from the Manuscript: The Methods section (lines 252–260) describes a detailed PCR and RT-PCR validation protocol, including breakpoint validation on DNA and gene expression validation on cDNA, with specific thermocycler conditions, primer concentrations, and gel electrophoresis parameters. This level of methodological detail implies that PCR validation results are available and will be presented.

Results: The scientific findings (lines 507–531) are exclusively about the BC986 strain from the **2023 cohort**, specifically: (1) truncation of *unc-36* at the chromosome III breakpoint of eT1(III;V), and (2) aberrant expression of *H14N18.2* near the chromosome V breakpoint. Both findings are supported only by visual IGV read-coverage tracks (Figure 4). No PCR or RT-PCR result is shown for either finding.

There seems to be a mismatch:

The gel shown is from the **2024 cohort**, but the scientific results are from the **2023 cohort**. The gel has no labels connecting it to any specific gene, strain, or finding. It therefore serves neither as validation of the *unc-36* truncation claim nor as confirmation of *H14N18.2* expression. It floats in the supplementary document as an illustration of classroom activity, which is fine pedagogically, but it does not meet the evidentiary standard for a scientific results section claiming a discovery.

This compounds the concern raised earlier: the manuscript describes PCR validation methods in enough detail to suggest the experiments were performed, but the only gel presented is unrelated to the findings being claimed, and no quantitative expression data is shown for the genes discussed.

The following two options can be considered for the revision:

Option A. Provide the relevant validation data. If PCR validation of *unc-36* truncation or *H14N18.2* expression was performed on BC986 (2023 cohort), those results should be shown with properly labeled gel lanes, appropriate controls (positive, negative, and no-template), and a figure legend that connects the gel to the specific claim being made. This would genuinely strengthen the scientific result.

Option B. Reframe the gel as pedagogical evidence only. If PCR validation was not performed for the specific genes discussed in the Results, the authors should remove the validation methods from the Methods section (or clearly restrict them to pedagogical context), acknowledge in the Results that the *unc-36* and *H14N18.2* findings have not yet been independently validated by PCR, and retain the gel image in the supplementary materials as evidence of the course's wet-lab component, explicitly labelled as such, not as scientific validation of the manuscript's claims.

Option B is more straightforward given what is currently in the manuscript. Presentation of a 2024 classroom gel as supplementary support for 2023 sequencing-based findings should be appropriately revised.

The Results and Methods sections overlap substantially

[Lines 262–496]

The Results section opens by verifying the course satisfies five CURE criteria, then proceeds through sub-sections on curriculum design, pedagogy, collaboration strategies, active learning activities, and student assessment, the majority of which are detailed course descriptions, not results. The actual results including the survey data and the eT1 scientific finding, appear only late in the section.

This structural problem means the paper's actual findings are buried after extensive methodological narration. A clearer organization would place course design details in the Methods and use the Results section exclusively for: (1) evidence of CURE criteria fulfillment (briefly), (2) survey-based student outcomes, and (3) scientific discoveries.

- Reorganizing the paper so that course design specifics (collaboration strategy, active learning activities, assignment scaffolding) are in the Methods, and the Results section focuses on measured or observed outcomes would strengthen the manuscript.

Conflict with Table 2

Line 210 reference: The text reads *"At each cycle of the course, three different C. elegans balancer strains were analyzed (Table 2)."* This calls for a table listing the balancer strains used across all three cohorts.

That table does not actually exist as a formatted table in the manuscript. Instead, the strain information is presented immediately afterward as a bulleted list (lines 214–216):

- 2023: BC986, BC4586, and VC109
- 2024: CZ1072, MT690, and SS746
- 2025: BC1217, GE2722, and RW6002

The "(Table 2)" citation at line 210 appears to be a placeholder reference that was never resolved — either the authors intended to format this information as a table and reverted to a list without removing the table call, or the table was present in an earlier draft and was deleted without updating the in-text citation.

Line 297: An entirely separate table, "Table 2: Bioinformatics core competencies", is formally numbered and presented. This is a properly formatted table.

The result is that the manuscript has no Table 2 where one is cited (line 210), and a different Table 2 exists where it is correctly cited (line 297). Every subsequent table number in the manuscript is therefore also potentially affected if the balancer strains table is reinstated.

Reference 69 should be replaced with Wilson Sayres et al. (2018)

[Lines 290–291]

The manuscript cites reference 69 (Gao & Guo, 2023) when introducing the NIBLSE bioinformatics core competencies framework. However, the primary, peer-reviewed source for this framework is the Wilson

Sayres et al. (2018) paper in PLoS ONE, which is the paper the NIBLSE community itself uses as the canonical reference for these competencies.

Reference: *Wilson Sayres MA, Hauser C, Sierk M, Robic S, Rosenwald AG, Smith TM, et al. Bioinformatics core competencies for undergraduate life sciences education. PLoS ONE. 2018;13(6):e0196878.*

The Gao & Guo (2023) paper may be retained as a secondary citation.

The Discussion is too brief and does not engage with limitations

[Lines 533–592]

At approximately 3 pages, the Discussion is disproportionately short relative to the complexity of what is being claimed. It does not seriously engage with the low survey response rate; the absence of control conditions; the heterogeneity of tools used across student teams; or the infrastructure requirements that may limit reproducibility at other institutions. It also opens (lines 534–552) by largely repeating the Introduction background rather than advancing the argument.

- Add a dedicated limitations section within the Discussion.
- Remove the repetition of background content from the opening of the Discussion and replace it with synthesis of the manuscript's findings in light of the cited literature.
- Discuss the scalability of the CURE design with greater clarity: what infrastructure is genuinely transferable (Lab Book, Galaxy-based analyses, open data), and what may not be (local HPC, *C. elegans* research group support, Research Coach funding).

Author contributions statement is absent

[Author list, Lines 4–11]

With more than 30 co-authors, PLoS ONE requires an individual author contributions statement. None is present. For a CURE manuscript where students are co-authors, this is particularly important: readers need to understand the criteria by which student participation qualified for authorship versus acknowledgment, and what each named individual specifically contributed. This is also a scholarly question of broader interest to the CURE community.

- Add a complete Author Contributions statement specifying each co-author's contribution using CRediT taxonomy roles (e.g., Data Curation, Formal Analysis, Investigation, Writing).
- Clarify the authorship criteria applied to student participants.

Minor and Editorial Comments

The following issues are correctable during revision. A thorough professional language edit is strongly recommended, as the manuscript shows systematic evidence of French-to-English translation artifacts throughout.

Systematic French-to-English interference errors

The manuscript seemingly is drafted partly in French. Several non-standard English constructions appear repeatedly and should be corrected in a targeted pass:

Current phrasing	Suggested revision

Line 149 — 'Yearly planification'	'Annual Schedule' or 'Course Timeline'
Line 112 — 'consists in the analysis'	'consists of' (preposition error)
Line 68 — 'it is then challenging to define'	'it is therefore difficult to define'
Line 29 — 'rare are the undergraduate programs'	'Few undergraduate programs offer'
Line 568 — 'the charge of work it might represent'	'the workload it may represent'
Line 589 — 'becomes then not only beneficial'	'is beneficial not only'
Line 546 — 'by "marketing" the courses'	'by improving how bioinformatics courses are framed and presented to prospective students'

Tense inconsistency in the Methods section

[Lines 118–260]

The Methods section alternates between present tense ('All materials are prepared,' 'TAs are recruited') and past tense ('The survey was accessible,' 'Students were guided') inconsistently. Course descriptions that apply generally across iterations can use present tense; specific actions taken during the 2023–2024 study period can use past tense. The authors should choose a convention and apply it consistently.

Specific typographical and grammatical errors

The following specific errors were identified:

- Line 245: 'Adapters er removed' → 'Adapters are removed'
- Line 305: 'Bachelor of Heath Sciences' → 'Bachelor of Health Sciences'
- Line 498: 'MDCS 301 CURE' → 'MDSC 301 CURE'
- Lines 290–291: Sentence reads 'students are familiar with MDSC 301 CURE' → should be 'students are familiar with through MDSC 301'
- Line 76: The trailing ellipsis in '(genomics, transcriptomics, metabolomics, proteomics…)' creates an incomplete parenthetical. Consider: 'including genomics, transcriptomics, metabolomics, and proteomics'
- Lines 378–379: 'Reports are individuals' is grammatically incorrect. Should read: 'Reports are individual written assignments in which questions are designed to prompt student reflection on their own performance and contribution to the team'.
- The word "un-diverse" (line 553) is not standard English and should be replaced with "homogeneous" or "lacking diversity." In the same passage, "Women and other minority groups are also dismal in the computer science professions" is grammatically incorrect; "dismal" inadvertently describes the groups rather than their representation; "the representation of women and minority groups remains alarmingly low" is more accurate.

Week 7 is absent from the course schedule without explanation

[Figure 2; Lines 151–159]

Figure 2 presents the 12-week semester schedule but jumps from Week 6 directly to Week 8. Week 7 does not appear in the figure and is not mentioned in the text. Given that the schedule is central to

understanding the course design, this gap is notable. If Week 7 is a spring break or buffer week, this should be stated. If it is a data entry omission, the figure should be corrected.

- Clarify what occurs in Week 7 in both the figure and the accompanying text.

Figure 3 is difficult to interpret

Figure 3 contains very small text in both panels, making the survey item labels nearly illegible in print. Panel B reports the percentage of respondents who said the experience 'improved' certain skills, but without a defined baseline or scale for 'magnitude of improvement,' the bars cannot be interpreted objectively. The predominantly positive framing of Likert items in Panel A also introduces acquiescence bias. Consider whether presenting individual response distributions (e.g., stacked bar with n labelled) would be more transparent given the small sample size.

- Increase font size in Figure 3 to make it more legible.
- Add the n value (9) as a note directly on the figure.
- Clarify in the caption what 'improved' means in Panel B and what scale participants used to rate improvement.

The title emphasizes the scientific question over the educational contribution

The current title — 'A Course-Undergraduate Research Experience (CURE) to explore the effect of structural variants on gene expression in *C. elegans* balancers' foregrounds the *C. elegans* genomics question rather than the educational innovation, which is the manuscript's primary contribution.

Suggested revision: *Consider a revised title such as: 'A bioinformatics CURE using C. elegans structural variant analysis as an authentic undergraduate research experience: design, implementation, and student outcomes'*

Final Remarks

This manuscript describes a genuinely valuable contribution to bioinformatics education. The CURE design is innovative, the open-science approach is exemplary, and the dual-purpose model as seen in the generation of real scientific discoveries through undergraduate coursework, is the kind of creative, high-impact work that the educational research community needs. These strengths make the concerns raised above worth addressing carefully, because the work deserves to be published in its strongest possible form.

The authors are encouraged to: (1) rigorously qualify all educational outcome claims to match the available evidence; (2) add quantitative analysis for the scientific finding; (3) undertake a thorough language edit with attention to the specific issues documented in the minor and editorial comments; and (4) restructure the Introduction and Results sections for cleaner logical flow. A well-revised manuscript addressing these concerns would make a strong and publishable contribution.
