# Supplementary material for: A course-undergraduate research experience (CURE) to explore the effect of structural variants on gene expression in C. elegans balancers
Source: PLoS One. 2026 Aug 11;21(8):e0342806. doi: 10.1371/journal.pone.0342806 (PMC13460597; doi:10.1371/journal.pone.0342806)
Supplement: Supplementary file 3 [file pone.0342806.s003.docx]

## Response to editor

Thank you for this contribution to PLOS One. All three reviewers agree that you describe an interesting and potentially useful CURE. As you note in the manuscript, this type of learning experience can be extremely valuable to our students. However, the reviewers also generally agreed that the manuscript should be revised. They express concerns about overstating the conclusions and the analysis of the data[MT1.1]. I hope you find their comments useful as you contemplate revising this manuscript.

We thank the editor for their comments and we are pleased to re-submit a manuscript where we have incorporated many changes to take into account both editor’s and reviewers’ comments. We addressed the major concerns regarding the overstating conclusions by reformulating such conclusion in the context of the data collected only.

## Response to reviewers

Reviewer #1: In the manuscript titled “A Course-Undergraduate Research Experience (CURE) to explore the effect of structural variants on gene expression in *C. elegans* balancers,” Maroilley et al. describe the development and implementation of a CURE that focuses on the teaching of bioinformatics through an applied and inquiry-based approach. The authors developed a substantial number of materials to support the implementation of the CURE in the course MDSC 301. The course appears to be well thought-out and well-scaffolded to guide the students through the challenging material. Many undergraduate curriculums lack quality courses in bioinformatics; therefore, there is a clear value to this course and the approach the authors are taking to introduce this topic to their students.

Unfortunately, the assessment of the learning outcomes and value of the course to the students is very limited. Given that this manuscript was submitted as a research article, there does not appear to be enough robust analysis of the course to warrant it in this category**.** My recommendation would be for the authors to consider submitting the manuscript to an education journal, focusing on their pedagogical approach to the course.

We thank Reviewer 1 for their detailed comments and suggestions on our manuscript that we addressed in a revied manuscript.

Major Concerns:

• The actual assessment of the course is only described in a few sentences (lines 498-503) and is very limited in scope. The response rate of the survey was only 13% (9 students), which is unfortunately too low to obtain any substantial feedback. A higher response rate and a different experimental design would allow for more robust and informative assessment of the outcomes of the course for the students.
- These 9 students come from two separate cohorts. What was changed in the course from cohort 1 vs. cohort 2? Did this have an impact on the outcomes for the students?

The main changes between cohort 1 and cohort 2 were changes regarding the schedule to accommodate the projects implementations around the Winter break. In addition, students were introduced to R in a one-hour lecture and a one-hour activity session. Following students’ feedback and because ultimately, when given the choice, all groups chose to analyze their RNA-Seq data with Galaxy, we chose to remove the R introduction the following years. Finally, in 2024 we acquired a portable PCR machine that allowed students to perform experimental validation of their findings (cohort 2). We have added description of these changes in the manuscript. In terms of the response rates, these align well with the published literature (5% to 30%) on response rates when these are made optional and consent is involved in higher education, presumably due to survey fatigue. We have now noted this in the manuscript and hope that the reviewers will agree that despite these shortcomings the data we gathered still provide valuable insights into the context of the data we collected.

- Did the impact of the course on the students vary by their major or educational background?

Unfortunately, the survey was anonymous, so we do not have access to educational background information.

- Best practice would involve having both a pre-survey and post-survey, so that you can assess the growth of the students throughout the course. The authors only provided a post-assessment to evaluate the learning objectives and outcomes of the course. While the students were given an initial survey in the course and reflections throughout, these were not designed as assessment tools.

Even though we agree with the reviewer, this is not how the CURE evaluation project was designed at UCalgary. We do not have the pre-survey. However, this is an excellent suggestion that will be communicated to UCalgary for future considerations.

- Best practice would involve having the students complete the survey as part of the course (their participation in the research study would still be optional). This should result in a much higher response rate. Additionally, cohort 1 was assessed almost one year after they completed the course (course in winter 2023, assessment in winter 2024).

This is not how the ethics were submitted and the project designed. It was voluntary and anonymous. However, these suggestions made by the reviewers have been communicated to the UCalgary for their consideration for futures studies.

- Even better – Incorporate some of the reflection assignments into your assessment strategy (with approval from IRB and consent from the students).

Assignments were originally designed as educational tools and ways to gather informal feedback for the instruction team to improve the curriculum. We agree with the reviewers that these feedbacks are valuable, but as the reviewer mentioned, we do not have IRB and consent to diffuse them. It would be close to impossible to recontact and consent the students now and uncertain of how valuable considering that these students have taken the class several years ago.

**We discuss now these very interesting points in the manuscript (see Discussion) (lines 585-600).**

“A post-course survey indicated that students’ interest and understanding in the field have improved. However, we acknowledge that the low participation in the survey (14% of students enrolled in Winters 2023 and 2024), is a limitation to our study and may not be applicable to all students [75]. Furthermore, considering that the survey was anonymous, we did not ask for any identifiable information such as ethnicity, major, or experiences. Therefore, we cannot consider for disparities of students’ experiences based on their background. In addition, survey’s participation was voluntary, and it was released after the grades were submitted, to avoid perception that the survey could affect students’ grades. Considering this, we were only left with a possibility to administer survey via online form, which could have affected the participation rate due to digital survey fatigue [77,78]. This may explain lower response rate as students often rapidly move on after the end of the Winter semester to new endeavors and might not be motivated to look back or may not even regularly check their student email boxes. However, the data we gathered still provides valuable insights. Future work would benefit from pre- and post-surveys to assess the growth of the students throughout the course [76]. Also, surveys could be part of the course but with participation in the research study still optional. In addition, reflection assignments could be included in the assessment strategy with the appropriate approval from IRB and consent from the students. **”**

- Although the course was implemented three times, only the outcomes of the first two cohorts were assessed.

Due to schedule reasons (the main instructor completed their postdoctoral training moved on), the course was assessed only twice.

- Based on the results shown in Figure 3, the only responses the students could provide were “strongly agree”, “somewhat agree”, or “neither agree or disagree”. Were students given the option to “somewhat disagree” and “strongly disagree”?

Students were given these options, but they were not picked by any of them. So, as an attempt to improve readability of the figure, we removed them. However, we have now added this information to the legend of the figures to improve clarity of the communication of our results.

• The figures lack figure legends. They would benefit from stronger titles and detailed explanations.

We have completed the legends of the figures.

• Figure 3A.
- As mentioned previously, did you allow students to “disagree” with the statements?

Yes, and we have added a note about it in the legend of the figure. The students were given five choices including “somewhat disagree” and “strongly disagree”, but because these two answers were never selected by any of the nine participants, we removed it from the bar plot to increase readability.

- The order of the statements in this figure seems random. It does not match the order of the questions in the survey, nor is it organized by the degree to which students agree with the statements or by topic.

We re-organized the statements according to the order of the questions in the survey.

- What do the numbers at the bottom represent (I assume number of students that responded?)

They represent the number of participants who selected a specific answer. We added the information in the Figure legend.

• Figure 3B.
- What do the numbers at the bottom represent? I assume % of students that somewhat or strongly agreed?

These data come from Q12 of the survey where participants could pick none, one or multiple answers. The numbers represent the % of participants who pick each answer. We have added this information to the legend.

- Again, the order of these statements seems random.

We have reordered the statements to follow the order in the survey.

Additional Comments:

• The manuscript would benefit from additional information on the background of the students that took this course. Was the course required for all students? Was it one of several elective courses? What prior lab experience did the students have prior to taking this course?
The course was not required for all students, only students enrolled in the bioinformatics program (only a third of the cohorts) need this class as a requirement. Prior requirements were 6 units in Computer Science at the 300 level or Medical Science 341, or 6 units in Biological Sciences at the 300 level. The course was not selective with 45 student capacity. We have added this information to the manuscript (lines 123-125).

• You mentioned that you alter the course and Lab Book each semester, based on feedback from the students, instructor, and TAs (lines 188-190). What are some examples of how you have revised the Lab Book and course?

We expanded on the changes in the manuscript (lines 215-226).

“Every year, one of the main alterations of the Lab book is related to calendar constraints. Indeed, we found it important to adjust the schedule around the week of the Winter break to avoid any major disruptions of the training project. In addition, during the first iteration, we had a one-hour lecture and a one-hour activity to present R to the students so they could choose to use either Galaxy or R to implement their RNA-Seq analysis. But students with no prior programming experience struggled and reported that it was challenging to grasp anything in such a short time. Ultimately, all groups chose to use Galaxy. The following years, we decided to remove the Introduction to R but still allow students with prior experience to use it for their project. The last major change was related to the acquisition of a portable PCR machine (miniPCR bio) that allowed the students to try out PCR during the genomics project to validate breakpoints during the second iteration. We added a session of validation of changes in gene expression via RT-PCR with the same equipment during the third iteration..”

• The way some portions of this manuscript were written was a bit unusual. It felt like some of the sentences were truncated or missing words. I think it would benefit from additional editing.

We have now edited the manuscript and revised the sentences.

• Lines 121-123: You describe that the course has been offered to three cohorts of students. However, you only provide survey results for two of these cohorts.

For time constraint reasons, we only distributed the survey to students twice.

• Lines 412-422. Walking Gallery. This section is not clear. I am not sure what they are doing in this part of the course and its objective.

We meant “Gallery walk” activity which is a recognized active teaching method. We clarified that section and added references (lines 338-348).

“2.	Gallery Walk: For students to acquire the necessary scientific knowledge to conduct a genomics analysis of SVs, we implemented a gallery walk activity. Previous research shows that this type of activity promotes discussion and facilitates knowledge acquisition [52–54]. Prior to the session, students were asked to read pre-selected articles (four per group, so one per student) in the light of the themes they would have to discuss during the activity (themes available in the Lab Book). In class, students went in groups from one board to another dedicated to a specific theme . Students were asked to write relevant information for the theme the board (e.g., Sequencing technologies” or “Biases in RNA-Seq”). Every ten minutes, each group walked towards the next board and built upon what was already written by previous groups. This activity was implemented twice: once for genomics and once for transcriptomics.”

• Lines 440-442: What do you mean that the presentation of the posters is “not subject to any evaluation for the students’ participation”? How do you know that the students understand the project and their results, and that they are meeting the learning objectives of the course? Even at professional conferences, presenters are challenged with their thinking and asked questions about their project. Plus, how do students learn if they don’t receive feedback? The experience can be positive and encouraging and still provide constructive feedback so that the students can improve in the future.

The understanding of the project is evaluated at every step during the project, by quizzes for background knowledge, and research-type group assignments. What we mean here is that we choose to mark the poster and its design but not their participation at the conference so that they can fully enjoy the experience and the opportunity to present and discuss their work with participants without the pression of a mark, just like in professional conferences indeed. We clarified that section (lines 366-369).

• Lines 557-560. What is the evidence that you have created an inclusive learning environment in this course and that students are supported in their learning? While I am sure you aim for these objectives, you need to provide evidence to support this statement.

We reformulate this statement. Our intention was to explain that we intended to provide an inclusive and supportive learning environment.

**Reviewer #2:** Overall, this is an excellent CURE and I believe that other educators will greatly benefit from following this example. With that being said, I do have a few comments regarding the overall structure and flow of the manuscript.

We thank Reviewer 2 for their comments and suggestions.

Remove the term "nowadays" found on lines 26 and 66. This is an unnecessary filler term.

Done

In your introduction only very briefly defines what a CURE is and there is no decision on even the hallmarks of a CURE until lines 264-287 in your results section. These defined hallmarks should be discussed in the introduction. That way your reader knowns exactly how a CURE is defined by the literature.

We added information on CURE lines 49-59.

Lines 263-496 (all found in the results section) should be moved out of the results section. This is the heart of your course design. If you are keeping with the traditional introduction, methods, results, discussion this this course design should be moved to the methods section with a subsection labeled as Course Design. These are not results but rather all of the important information on how the course was set up and executed.

We moved some of the descriptive paragraphs in the Methods section as suggested. We kept in the Results section the paragraphs bringing an analysis of the design of the course.

Line 147-148 there is a survey mentioned. This is confusing because there has been no mention of this survey and it is not brought up again until the Survey and Ethics statement starting on line 196. To help with the flow the Survey and Ethics Statement subsection (line 196-207) should be moved and place before the CURE Implementation subsection (line 140-148).

We thank Reviewer 2 for this comment. We change the manuscript accordingly.

The best approach at this point would be a reorganization of the paper in order to help with the flow and to make it seem less disjointed. It appears the overall focus is the design of the course so that should be made more clear in the introduction and the methods should be dedicated to the course design rather than the results section.

We have added more information at the end of the Introduction to better inform the reader regarding the content of the manuscript.

**Reviewer #3:** The paper addresses a genuinely important gap: the integration of authentic, original research into undergraduate bioinformatics education through a Course-based Undergraduate Research Experience (CURE). The course design is creative, well-scaffolded, and grounded in established CURE pedagogy. The commitment to open science via sharing the Lab Book, datasets, and protocols publicly is exemplary and adds considerable value for the broader community of bioinformatics educators.

We thank Reviewer 3 for their comments.

However, the manuscript in its current form requires major revision before it can be accepted. The concerns are concentrated in three areas:

(1) the educational outcome claims substantially overreach the data, which rests on a critically small and self-selected survey sample;

We have reviewed our statement and explained better the context in an attempt to avoid over-interpretation.

(2) the scientific discovery component lacks the quantitative analysis necessary for publication as a research finding; and

We added experimental validation of the scientific results in supplementary information.

(3) the writing requires thorough language editing to address recurrent grammatical issues, sentence-level clarity, and narrative structure.

We have now edited the manuscript and revised it to correct for these issues.
