# Supplementary material for: A course-undergraduate research experience (CURE) to explore the effect of structural variants on gene expression in C. elegans balancers
Source: PLoS One. 2026 Aug 11;21(8):e0342806. doi: 10.1371/journal.pone.0342806 (PMC13460597; doi:10.1371/journal.pone.0342806)
Supplement: Peer review history

# Peer review: Decision Letter 0

**DOI:** 10.1371/journal.pone.0342806.r001

**Authors:** David R Wessner

8 Apr 2026

Dear Dr. Maroilley,

Thank you for submitting your manuscript to PLOS ONE. After careful consideration, we feel that it has merit but does not fully meet PLOS ONE’s publication criteria as it currently stands. Therefore, we invite you to submit a revised version of the manuscript that addresses the points raised during the review process.

Please submit your revised manuscript by May 23 2026 11:59PM. If you will need more time than this to complete your revisions, please reply to this message or contact the journal office at plosone@plos.org. When you're ready to submit your revision, log on to https://www.editorialmanager.com/pone/ and select the 'Submissions Needing Revision' folder to locate your manuscript file.

- A letter that responds to each point raised by the academic editor and reviewer(s). You should upload this letter as a separate file labeled 'Response to Reviewers'.
- A marked-up copy of your manuscript that highlights changes made to the original version. You should upload this as a separate file labeled 'Revised Manuscript with Track Changes'.
- An unmarked version of your revised paper without tracked changes. You should upload this as a separate file labeled 'Manuscript'.

If you would like to make changes to your financial disclosure, please include your updated statement in your cover letter. Guidelines for resubmitting your figure files are available below the reviewer comments at the end of this letter.

If applicable, we recommend that you deposit your laboratory protocols in protocols.io to enhance the reproducibility of your results. Protocols.io assigns your protocol its own identifier (DOI) so that it can be cited independently in the future. For instructions see: https://journals.plos.org/plosone/s/submission-guidelines#loc-laboratory-protocols. Additionally, PLOS ONE offers an option for publishing peer-reviewed Lab Protocol articles, which describe protocols hosted on protocols.io. Read more information on sharing protocols at https://plos.org/protocols?utm_medium=editorial-email&utm_source=authorletters&utm_campaign=protocols.

As the corresponding author, your ORCID iD is verified in the submission system and will appear in the published article. PLOS supports the use of ORCID, and we encourage all coauthors to register for an ORCID iD and use it as well. Please encourage your coauthors to verify their ORCID iD within the submission system before final acceptance, as unverified ORCID iDs will not appear in the published article. *Only*  the individual author can complete the verification step; PLOS staff *cannot*  verify ORCID iDs on behalf of authors.

We look forward to receiving your revised manuscript.

Kind regards,

David R Wessner, Ph.D.

Academic Editor

PLOS One

Journal requirements:

When submitting your revision, we need you to address these additional requirements.

1. Please ensure that your manuscript meets PLOS ONE's style requirements, including those for file naming. The PLOS ONE style templates can be found at

https://journals.plos.org/plosone/s/file?id=wjVg/PLOSOne_formatting_sample_main_body.pdf and

https://journals.plos.org/plosone/s/file?id=ba62/PLOSOne_formatting_sample_title_authors_affiliations.pdf

2. We note that your consent statement reads "Consent was written, included at the beginning of the questionnaire". In the revised version of your manuscript could you please update this statement to reflect that informed consent was obtained?

If the reviewer comments include a recommendation to cite specific previously published works, please review and evaluate these publications to determine whether they are relevant and should be cited. There is no requirement to cite these works unless the editor has indicated otherwise.

Additional Editor Comments:

Thank you for this contribution to PLOS One. All three reviewers agree that you describe an interesting and potentially useful CURE. As you note in the manuscript, this type of learning experience can be extremely valuable to our students. However, the reviewers also generally agreed that the manuscript should be revised. They express concerns about overstating the conclusions and the analysis of the data. I hope you find their comments useful as you contemplate revising this manuscript.

[Note: HTML markup is below. Please do not edit.]

Reviewers' comments:

Reviewer's Responses to Questions

**Comments to the Author**

1. Is the manuscript technically sound, and do the data support the conclusions?

Reviewer #1: No

Reviewer #2: Yes

Reviewer #3: Partly

**********

2. Has the statistical analysis been performed appropriately and rigorously?

Reviewer #1: No

Reviewer #2: N/A

Reviewer #3: No

**********

3. Have the authors made all data underlying the findings in their manuscript fully available?

The PLOS Data policy

Reviewer #1: Yes

Reviewer #2: Yes

Reviewer #3: Yes

**********

4. Is the manuscript presented in an intelligible fashion and written in standard English?

Reviewer #1: Yes

Reviewer #2: Yes

Reviewer #3: No

**********

Reviewer #1: In the manuscript titled “A Course-Undergraduate Research Experience (CURE) to explore the effect of structural variants on gene expression in C. elegans balancers,” Maroilley et al. describe the development and implementation of a CURE that focuses on the teaching of bioinformatics through an applied and inquiry-based approach. The authors developed a substantial number of materials to support the implementation of the CURE in the course MDSC 301. The course appears to be well thought-out and well-scaffolded to guide the students through the challenging material. Many undergraduate curriculums lack quality courses in bioinformatics; therefore, there is a clear value to this course and the approach the authors are taking to introduce this topic to their students.

Unfortunately, the assessment of the learning outcomes and value of the course to the students is very limited. Given that this manuscript was submitted as a research article, there does not appear to be enough robust analysis of the course to warrant it in this category. My recommendation would be for the authors to consider submitting the manuscript to an education journal, focusing on their pedagogical approach to the course.

Major Concerns:

• The actual assessment of the course is only described in a few sentences (lines 498-503) and is very limited in scope. The response rate of the survey was only 13% (9 students), which is unfortunately too low to obtain any substantial feedback. A higher response rate and a different experimental design would allow for more robust and informative assessment of the outcomes of the course for the students.

- These 9 students come from two separate cohorts. What was changed in the course from cohort 1 vs. cohort 2? Did this have an impact on the outcomes for the students?

- Did the impact of the course on the students vary by their major or educational background?

- Best practice would involve having both a pre-survey and post-survey, so that you can assess the growth of the students throughout the course. The authors only provided a post-assessment to evaluate the learning objectives and outcomes of the course. While the students were given an initial survey in the course and reflections throughout, these were not designed as assessment tools.

- Best practice would involve having the students complete the survey as part of the course (their participation in the research study would still be optional). This should result in a much higher response rate. Additionally, cohort 1 was assessed almost one year after they completed the course (course in winter 2023, assessment in winter 2024).

- Even better – Incorporate some of the reflection assignments into your assessment strategy (with approval from IRB and consent from the students).

- Although the course was implemented three times, only the outcomes of the first two cohorts were assessed.

- Based on the results shown in Figure 3, the only responses the students could provide were “strongly agree”, “somewhat agree”, or “neither agree or disagree”. Were students given the option to “somewhat disagree” and “strongly disagree”?

• The figures lack figure legends. They would benefit from stronger titles and detailed explanations.

• Figure 3A.

- As mentioned previously, did you allow students to “disagree” with the statements?

- The order of the statements in this figure seems random. It does not match the order of the questions in the survey, nor is it organized by the degree to which students agree with the statements or by topic.

- What do the numbers at the bottom represent (I assume number of students that responded?)

• Figure 3B.

- What do the numbers at the bottom represent? I assume % of students that somewhat or strongly agreed?

- Again, the order of these statements seems random.

Additional Comments:

• The manuscript would benefit from additional information on the background of the students that took this course. Was the course required for all students? Was it one of several elective courses? What prior lab experience did the students have prior to taking this course?

• You mentioned that you alter the course and Lab Book each semester, based on feedback from the students, instructor, and TAs (lines 188-190). What are some examples of how you have revised the Lab Book and course?

• The way some portions of this manuscript were written was a bit unusual. It felt like some of the sentences were truncated or missing words. I think it would benefit from additional editing.

• Lines 121-123: You describe that the course has been offered to three cohorts of students. However, you only provide survey results for two of these cohorts.

• Lines 412-422. Walking Gallery. This section is not clear. I am not sure what they are doing in this part of the course and its objective.

• Lines 440-442: What do you mean that the presentation of the posters is “not subject to any evaluation for the students’ participation”? How do you know that the students understand the project and their results, and that they are meeting the learning objectives of the course? Even at professional conferences, presenters are challenged with their thinking and asked questions about their project. Plus, how do students learn if they don’t receive feedback? The experience can be positive and encouraging and still provide constructive feedback so that the students can improve in the future.

• Lines 557-560. What is the evidence that you have created an inclusive learning environment in this course and that students are supported in their learning? While I am sure you aim for these objectives, you need to provide evidence to support this statement.

Reviewer #2: Overall, this is an excellent CURE and I believe that other educators will greatly benefit from following this example. With that being said, I do have a few comments regarding the overall structure and flow of the manuscript.

Remove the term "nowadays" found on lines 26 and 66. This is an unnecessary filler term.

In your introduction only very briefly defines what a CURE is and there is no decision on even the hallmarks of a CURE until lines 264-287 in your results section. These defined hallmarks should be discussed in the introduction. That way your reader knowns exactly how a CURE is defined by the literature.

Lines 263-496 (all found in the results section) should be moved out of the results section. This is the heart of your course design. If you are keeping with the traditional introduction, methods, results, discussion this this course design should be moved to the methods section with a subsection labeled as Course Design. These are not results but rather all of the important information on how the course was set up and executed.

Line 147-148 there is a survey mentioned. This is confusing because there has been no mention of this survey and it is not brought up again until the Survey and Ethics statement starting on line 196. To help with the flow the Survey and Ethics Statement subsection (line 196-207) should be moved and place before the CURE Implementation subsection (line 140-148).

The best approach at this point would be a reorganization of the paper in order to help with the flow and to make it seem less disjointed. It appears the overall focus is the design of the course so that should be made more clear in the introduction and the methods should be dedicated to the course design rather than the results section.

Reviewer #3: The paper addresses a genuinely important gap: the integration of authentic, original research into undergraduate bioinformatics education through a Course-based Undergraduate Research Experience (CURE). The course design is creative, well-scaffolded, and grounded in established CURE pedagogy. The commitment to open science via sharing the Lab Book, datasets, and protocols publicly is exemplary and adds considerable value for the broader community of bioinformatics educators.

However, the manuscript in its current form requires major revision before it can be accepted. The concerns are concentrated in three areas: (1) the educational outcome claims substantially overreach the data, which rests on a critically small and self-selected survey sample; (2) the scientific discovery component lacks the quantitative analysis necessary for publication as a research finding; and (3) the writing requires thorough language editing to address recurrent grammatical issues, sentence-level clarity, and narrative structure.

**********

what does this mean?). If published, this will include your full peer review and any attached files.

If you choose “no”, your identity will remain anonymous but your review may still be made public.

**Do you want your identity to be public for this peer review?**  For information about this choice, including consent withdrawal, please see our Privacy Policy

Reviewer #1: No

Reviewer #2: No

Reviewer #3: No

**********

[NOTE: If reviewer comments were submitted as an attachment file, they will be attached to this email and accessible via the submission site. Please log into your account, locate the manuscript record, and check for the action link "View Attachments". If this link does not appear, there are no attachment files.]

To ensure your figures meet our technical requirements, please review our figure guidelines: https://journals.plos.org/plosone/s/figures

You may also use PLOS’s free figure tool, NAAS, to help you prepare publication quality figures: https://journals.plos.org/plosone/s/figures#loc-tools-for-figure-preparation.

NAAS will assess whether your figures meet our technical requirements by comparing each figure against our figure specifications.

Attachment Submitted filename: PONE-D-25-66689_ReviewerComments.pdf

# Author comment: Author response to Decision Letter 1

**DOI:** 10.1371/journal.pone.0342806.r002

29 May 2026

Response to editor

Thank you for this contribution to PLOS One. All three reviewers agree that you describe an interesting and potentially useful CURE. As you note in the manuscript, this type of learning experience can be extremely valuable to our students. However, the reviewers also generally agreed that the manuscript should be revised. They express concerns about overstating the conclusions and the analysis of the data[MT1.1]. I hope you find their comments useful as you contemplate revising this manuscript.

We thank the editor for their comments and we are pleased to re-submit a manuscript where we have incorporated many changes to take into account both editor’s and reviewers’ comments. We addressed the major concerns regarding the overstating conclusions by reformulating such conclusion in the context of the data collected only.

Response to reviewers

Reviewer #1: In the manuscript titled “A Course-Undergraduate Research Experience (CURE) to explore the effect of structural variants on gene expression in C. elegans balancers,” Maroilley et al. describe the development and implementation of a CURE that focuses on the teaching of bioinformatics through an applied and inquiry-based approach. The authors developed a substantial number of materials to support the implementation of the CURE in the course MDSC 301. The course appears to be well thought-out and well-scaffolded to guide the students through the challenging material. Many undergraduate curriculums lack quality courses in bioinformatics; therefore, there is a clear value to this course and the approach the authors are taking to introduce this topic to their students.

Unfortunately, the assessment of the learning outcomes and value of the course to the students is very limited. Given that this manuscript was submitted as a research article, there does not appear to be enough robust analysis of the course to warrant it in this category. My recommendation would be for the authors to consider submitting the manuscript to an education journal, focusing on their pedagogical approach to the course.

We thank Reviewer 1 for their detailed comments and suggestions on our manuscript that we addressed in a revied manuscript.

Major Concerns:

• The actual assessment of the course is only described in a few sentences (lines 498-503) and is very limited in scope. The response rate of the survey was only 13% (9 students), which is unfortunately too low to obtain any substantial feedback. A higher response rate and a different experimental design would allow for more robust and informative assessment of the outcomes of the course for the students.

- These 9 students come from two separate cohorts. What was changed in the course from cohort 1 vs. cohort 2? Did this have an impact on the outcomes for the students?

The main changes between cohort 1 and cohort 2 were changes regarding the schedule to accommodate the projects implementations around the Winter break. In addition, students were introduced to R in a one-hour lecture and a one-hour activity session. Following students’ feedback and because ultimately, when given the choice, all groups chose to analyze their RNA-Seq data with Galaxy, we chose to remove the R introduction the following years. Finally, in 2024 we acquired a portable PCR machine that allowed students to perform experimental validation of their findings (cohort 2). We have added description of these changes in the manuscript. In terms of the response rates, these align well with the published literature (5% to 30%) on response rates when these are made optional and consent is involved in higher education, presumably due to survey fatigue. We have now noted this in the manuscript and hope that the reviewers will agree that despite these shortcomings the data we gathered still provide valuable insights into the context of the data we collected.

- Did the impact of the course on the students vary by their major or educational background?

Unfortunately, the survey was anonymous, so we do not have access to educational background information.

- Best practice would involve having both a pre-survey and post-survey, so that you can assess the growth of the students throughout the course. The authors only provided a post-assessment to evaluate the learning objectives and outcomes of the course. While the students were given an initial survey in the course and reflections throughout, these were not designed as assessment tools.

Even though we agree with the reviewer, this is not how the CURE evaluation project was designed at UCalgary. We do not have the pre-survey. However, this is an excellent suggestion that will be communicated to UCalgary for future considerations.

- Best practice would involve having the students complete the survey as part of the course (their participation in the research study would still be optional). This should result in a much higher response rate. Additionally, cohort 1 was assessed almost one year after they completed the course (course in winter 2023, assessment in winter 2024).

This is not how the ethics were submitted and the project designed. It was voluntary and anonymous. However, these suggestions made by the reviewers have been communicated to the UCalgary for their consideration for futures studies.

- Even better – Incorporate some of the reflection assignments into your assessment strategy (with approval from IRB and consent from the students).

Assignments were originally designed as educational tools and ways to gather informal feedback for the instruction team to improve the curriculum. We agree with the reviewers that these feedbacks are valuable, but as the reviewer mentioned, we do not have IRB and consent to diffuse them. It would be close to impossible to recontact and consent the students now and uncertain of how valuable considering that these students have taken the class several years ago.

We discuss now these very interesting points in the manuscript (see Discussion) (lines 585-600).

“A post-course survey indicated that students’ interest and understanding in the field have improved. However, we acknowledge that the low participation in the survey (14% of students enrolled in Winters 2023 and 2024), is a limitation to our study and may not be applicable to all students [75]. Furthermore, considering that the survey was anonymous, we did not ask for any identifiable information such as ethnicity, major, or experiences. Therefore, we cannot consider for disparities of students’ experiences based on their background. In addition, survey’s participation was voluntary, and it was released after the grades were submitted, to avoid perception that the survey could affect students’ grades. Considering this, we were only left with a possibility to administer survey via online form, which could have affected the participation rate due to digital survey fatigue [77,78]. This may explain lower response rate as students often rapidly move on after the end of the Winter semester to new endeavors and might not be motivated to look back or may not even regularly check their student email boxes. However, the data we gathered still provides valuable insights. Future work would benefit from pre- and post-surveys to assess the growth of the students throughout the course [76]. Also, surveys could be part of the course but with participation in the research study still optional. In addition, reflection assignments could be included in the assessment strategy with the appropriate approval from IRB and consent from the students. ”

- Although the course was implemented three times, only the outcomes of the first two cohorts were assessed.

Due to schedule reasons (the main instructor completed their postdoctoral training moved on), the course was assessed only twice.

- Based on the results shown in Figure 3, the only responses the students could provide were “strongly agree”, “somewhat agree”, or “neither agree or disagree”. Were students given the option to “somewhat disagree” and “strongly disagree”?

Students were given these options, but they were not picked by any of them. So, as an attempt to improve readability of the figure, we removed them. However, we have now added this information to the legend of the figures to improve clarity of the communication of our results.

• The figures lack figure legends. They would benefit from stronger titles and detailed explanations.

We have completed the legends of the figures.

• Figure 3A.

- As mentioned previously, did you allow students to “disagree” with the statements?

Yes, and we have added a note about it in the legend of the figure. The students were given five choices including “somewhat disagree” and “strongly disagree”, but because these two answers were never selected by any of the nine participants, we removed it from the bar plot to increase readability.

- The order of the statements in this figure seems random. It does not match the order of the questions in the survey, nor is it organized by the degree to which students agree with the statements or by topic.

We re-organized the statements according to the order of the questions in the survey.

- What do the numbers at the bottom represent (I assume number of students that responded?)

They represent the number of participants who selected a specific answer. We added the information in the Figure legend.

• Figure 3B.

- What do the numbers at the bottom represent? I assume % of students that somewhat or strongly agreed?

These data come from Q12 of the survey where participants could pick none, one or multiple answers. The numbers represent the % of participants who pick each answer. We have added this information to the legend.

- Again, the order of these statements seems random.

We have reordered the statements to follow the order in the survey.

Additional Comments:

• The manuscript would benefit from additional information on the background of the students that took this course. Was the course required for all students? Was it one of several elective courses? What prior lab experience did the students have prior to taking this course?

The course was not required for all students, only students enrolled in the bioinformatics program (only a third of the cohorts) need this class as a requirement. Prior requirements were 6 units in Computer Science at the 300 level or Medical Science 341, or 6 units in Biological Sciences at the 300 level. The course was not selective with 45 student capacity. We have added this information to the manuscript (lines 123-125).

• You mentioned that you alter the course and Lab Book each semester, based on feedback from the students, instructor, and TAs (lines 188-190). What are some examples of how you have revised the Lab Book and course?

We expanded on the changes in the manuscript (lines 215-226).

“Every year, one of the main alterations of the Lab book is related to calendar constraints. Indeed, we found it important to adjust the schedule around the week of the Winter break to avoid any major disruptions of the training project. In addition, during the first iteration, we had a one-hour lecture and a one-hour activity to present R to the students so they could choose to use either Galaxy or R to implement their RNA-Seq analysis. But students with no prior programming experience struggled and reported that it was challenging to grasp anything in such a short time. Ultimately, all groups chose to use Galaxy. The following years, we decided to remove the Introduction to R but still allow students with prior experience to use it for their project. The last major change was related to the acquisition of a portable PCR machine (miniPCR bio) that allowed the students to try out PCR during the genomics project to validate breakpoints during the second iteration. We added a session of validation of changes in gene expression via RT-PCR with the same equipment during the third iteration..”

• The way some portions of this manuscript were written was a bit unusual. It felt like some of the sentences were truncated or missing words. I think it would benefit from additional editing.

We have now edited the manuscript and revised the sentences.

• Lines 121-123: You describe that the course has been offered to three cohorts of students. However, you only provide survey results for two of these cohorts.

For time constraint reasons, we only distributed the survey to students twice.

• Lines 412-422. Walking Gallery. This section is not clear. I am not sure what they are doing in this part of the course and its objective.

We meant “Gallery walk” activity which is a recognized active teaching method. We clarified that section and added references (lines 338-348).

“2. Gallery Walk: For students to acquire the necessary scientific knowledge to conduct a genomics analysis of SVs, we implemented a gallery walk activity. Previous research shows that this type of activity promotes discussion and facilitates knowledge acquisition [52–54]. Prior to the session, students were asked to read pre-selected articles (four per group, so one per student) in the light of the themes they would have to discuss during the activity (themes available in the Lab Book). In class, students went in groups from one board to another dedicated to a specific theme . Students were asked to write relevant information for the theme the board (e.g., Sequencing technologies” or “Biases in RNA-Seq”). Every ten minutes, each group walked towards the next board and built upon what was already written by previous groups. This activity was implemented twice: once for genomics and once for transcriptomics.”

• Lines 440-442: What do you mean that the presentation of the posters is “not subject to any evaluation for the students’ participation”? How do you know that the students understand the project and their results, and that they are meeting the learning objectives of the course? Even at professional conferences, presenters are challenged with their thinking and asked questions about their project. Plus, how do students learn if they don’t receive feedback? The experience can be positive and encouraging and still provide constructive feedback so that the students can improve in the future.

The understanding of the project is evaluated at every step during the project, by quizzes for background knowledge, and research-type group assignments. What we mean here is that we choose to mark the poster and its design but not their participation at the conference so that they can fully enjoy the experience and the opportunity to present and discuss their work with participants without the pression of a mark, just like in professional conferences indeed. We clarified that section (lines 366-369).

• Lines 557-560. What is the evidence that you have created an inclusive learning environment in this course and that students are supported in their learning? While I am sure you aim for these objectives, you need to provide evidence to support this statement.

We reformulate this statement. Our intention was to explain that we intended to provide an inclusive and supportive learning environment.

Reviewer #2: Overall, this is an excellent CURE and I believe that other educators will greatly benefit from following this example. With that being said, I do have a few comments regarding the overall structure and flow of the manuscript.

We thank Reviewer 2 for their comments and suggestions.

Remove the term "nowadays" found on lines 26 and 66. This is an unnecessary filler term.

Done

In your introduction only very briefly defines what a CURE is and there is no decision on even the hallmarks of a CURE until lines 264-287 in your results section. These defined hallmarks should be discussed in the introduction. That way your reader knowns exactly how a CURE is defined by the literature.

We added information on CURE lines 49-59.

Lines 263-496 [MT1.1](all found in the results section) should be moved out of the results section. This is the heart of your course design. If you are keeping with the traditional introduction, methods, results, discussion this this course design should be moved to the methods section with a subsection labeled as Course Design. These are not results but rather all of

Attachment Submitted filename: Response_to_Reviewers_REVISIONS.docx

# Peer review: Decision Letter 1

**DOI:** 10.1371/journal.pone.0342806.r003

**Authors:** David R Wessner

23 Jul 2026

A Course-Undergraduate Research Experience (CURE) to explore the effect of structural variants on gene expression in C. elegans balancers

PONE-D-25-66689R1

Dear Dr. Maroilley,

We’re pleased to inform you that your manuscript has been judged scientifically suitable for publication and will be formally accepted for publication once it meets all outstanding technical requirements.

Within one week, you’ll receive an e-mail detailing the required amendments. When these have been addressed, you’ll receive a formal acceptance letter and your manuscript will be scheduled for publication.

An invoice will be generated when your article is formally accepted. Please note, if your institution has a publishing partnership with PLOS and your article meets the relevant criteria, all or part of your publication costs will be covered. Please make sure your user information is up-to-date by logging into Editorial Manager at Editorial Manager® and clicking the ‘Update My Information' link at the top of the page. For questions related to billing, please contact billing support.

If your institution or institutions have a press office, please notify them about your upcoming paper to help maximize its impact. If they’ll be preparing press materials, please inform our press team as soon as possible -- no later than 48 hours after receiving the formal acceptance. Your manuscript will remain under strict press embargo until 2 pm Eastern Time on the date of publication. For more information, please contact onepress@plos.org.

Kind regards,

David R Wessner, Ph.D.

Academic Editor

PLOS One

Additional Editor Comments (optional):

Thank you so much for submitting this revised mansucript. The reviewers agreed that you addressed their original concerns. As a result, I would like to accept this mansucript for publication. It will be a nice addition to the literature. One reviewer did note some minor issues (grammar, mostly) that shoud be addressed. I included them below. Please make these changes before before the work is published. Thanks again for submitting htis manuscript to PLOS One.

Reviwer comments:

1. The authors regularly switch between past and present tense. For example, lines 77-84, lines 288-296, and lines 331-339. Other small grammar and clarity issues are also present throughout the text. For example, lines 398-401 are not clear. Line 395, "the teaching time talked..."

2. All abbreviations/acronyms need to be defined. Line 95 (WGS) and line 114 (BHSc).

3. Line 115, you say that the CURE was implemented 4 times. Line 132-133, you say it was 3 times, but then list 4 semesters. Which is correct?

4. Line 131, you say the course has a 45-student capacity, but then in line 133, you say it accommodated up to 44 students each semester. I know what you are trying to say, but it ends up being a bit confusing with the way it is written.

Reviewers' comments:

Reviewer's Responses to Questions

**Comments to the Author**

Reviewer #1: (No Response)

Reviewer #2: All comments have been addressed

**********

2. Is the manuscript technically sound, and do the data support the conclusions?

Reviewer #1: Yes

Reviewer #2: Yes

**********

3. Has the statistical analysis been performed appropriately and rigorously?

Reviewer #1: Yes

Reviewer #2: Yes

**********

4. Have the authors made all data underlying the findings in their manuscript fully available?

The PLOS Data policy

Reviewer #1: Yes

Reviewer #2: Yes

**********

5. Is the manuscript presented in an intelligible fashion and written in standard English?

Reviewer #1: Yes

Reviewer #2: Yes

**********

Reviewer #1: The revised manuscript is greatly improved from the first submission. However, a few minor issues should still be addressed:

1. While the grammar and writing quality is better, it still would benefit from additional improvement. In particular, the authors regularly switch between past and present tense. For example, lines 77-84, lines 288-296, and lines 331-339. Other small grammar and clarity issues are also present throughout the text. For example, lines 398-401 are not clear. Line 395, "the teaching time talked..."

2. All abbreviations/acronyms need to be defined. Line 95 (WGS) and line 114 (BHSc).

3. Line 115, you say that the CURE was implemented 4 times. Line 132-133, you say it was 3 times, but then list 4 semesters. Which is correct?

4. Line 131, you say the course has a 45-student capacity, but then in line 133, you say it accommodated up to 44 students each semester. I know what you are trying to say, but it ends up being a bit confusing with the way it is written.

5. Line 551. Switching from a description of the CURE and its results to the description of the chromosomal translocation is abrupt. It would benefit from a sentence or two transition.

Reviewer #2: (No Response)

**********

what does this mean?). If published, this will include your full peer review and any attached files.

If you choose “no”, your identity will remain anonymous but your review may still be made public.

**Do you want your identity to be public for this peer review?**  For information about this choice, including consent withdrawal, please see our Privacy Policy

Reviewer #1: No

Reviewer #2: No

**********

# Editor report: Acceptance letter

**DOI:** 10.1371/journal.pone.0342806.r004

**Authors:** David R Wessner

PONE-D-25-66689R1

PLOS One

Dear Dr. Maroilley,

I'm pleased to inform you that your manuscript has been deemed suitable for publication in PLOS One. Congratulations! Your manuscript is now being handed over to our production team.

At this stage, our production department will prepare your paper for publication. This includes ensuring the following:

* All references, tables, and figures are properly cited

* All relevant supporting information is included in the manuscript submission,

* There are no issues that prevent the paper from being properly typeset

You will receive further instructions from the production team, including instructions on how to review your proof when it is ready. Please keep in mind that we are working through a large volume of accepted articles, so please give us a few days to review your paper and let you know the next and final steps.

Lastly, if your institution or institutions have a press office, please let them know about your upcoming paper now to help maximize its impact. If they'll be preparing press materials, please inform our press team within the next 48 hours. Your manuscript will remain under strict press embargo until 2 pm Eastern Time on the date of publication. For more information, please contact onepress@plos.org.

You will receive an invoice from PLOS for your publication fee after your manuscript has reached the completed accept phase. If you receive an email requesting payment before acceptance or for any other service, this may be a phishing scheme. Learn how to identify phishing emails and protect your accounts at https://explore.plos.org/phishing.

If we can help with anything else, please email us at customercare@plos.org.

Thank you for submitting your work to PLOS ONE and supporting open access.

Kind regards,

PLOS ONE Editorial Office Staff

on behalf of

Dr. David R Wessner

Academic Editor

PLOS One
